# Hidden Diversity in Honey Bee Gut Symbionts Detected by Single-Cell Genomics

**DOI:** 10.1371/journal.pgen.1004596

**Published:** 2014-09-11

**Authors:** Philipp Engel, Ramunas Stepanauskas, Nancy A. Moran

**Affiliations:** 1 Department of Ecology and Evolutionary Biology, Yale University, New Haven, Connecticut, United States of America; 2 Bigelow Laboratory for Ocean Sciences, East Boothbay, Maine, United States of America; MicroTrek Incorporated, United States of America

## Abstract

Microbial communities in animal guts are composed of diverse, specialized bacterial species, but little is known about how gut bacteria diversify to produce genetically and ecologically distinct entities. The gut microbiota of the honey bee, *Apis mellifera*, presents a useful model, because it consists of a small number of characteristic bacterial species, each showing signs of diversification. Here, we used single-cell genomics to study the variation within two species of the bee gut microbiota: *Gilliamella apicola* and *Snodgrassella alvi*. For both species, our analyses revealed extensive variation in intraspecific divergence of protein-coding genes but uniformly high levels of 16S rRNA similarity. In both species, the divergence of 16S rRNA loci appears to have been curtailed by frequent recombination within populations, while other genomic regions have continuously diverged. Furthermore, gene repertoires differ markedly among strains in both species, implying distinct metabolic capabilities. Our results show that, despite minimal divergence at 16S rRNA genes, *in situ* diversification occurs within gut communities and generates bacterial lineages with distinct ecological niches. Therefore, important dimensions of microbial diversity are not evident from analyses of 16S rRNA, and single cell genomics has potential to elucidate processes of bacterial diversification.

## Author Summary

Gut microbial communities are often complex, consisting of bacteria from divergent phyla as well as multiple strains of each of the constituent species. But because the composition of these communities is typically assessed using 16S rRNA analyses, little is known about genomic changes associated with *in situ* diversification of bacterial lineages in animal guts. We undertook a single-cell genomic approach to investigate the diversification within two species of the gut microbiota of honey bees. Each species exhibited a surprisingly high level of genomic variation, despite uniformity in the 16S rRNA sequences. Our data indicate that genetically and ecologically distinct lineages can evolve in the gut of the same host species in the presence of frequent recombination at 16S rRNA genes. These findings parallel observations from mammals, suggesting that *in situ* diversification of a few bacterial lineages is a common pattern in the evolution of gut communities.

## Introduction

Animals contain complex bacterial communities in their guts that can impact health status [1]–[3]. In mammals, the phylogenetic architecture of gut communities has been described as fan-like, with few deep- and intermediate-branching lineages, but with many shallow branches [3]. Most of these bacteria live exclusively in the gut environment, suggesting that phylogenetic clusters have evolved *in situ* through diversification of a few founder species.

Diversity of gut communities is typically assessed using deep-sequencing of 16S rRNA PCR amplicons [4], [5], often with the aim of illuminating community differences between closely related hosts or between hosts with different environments or diets [6]–[9]. To this end, 16S rRNA sequences are clustered into operational taxonomic units (OTUs), and a cutoff of 97–98% identity is applied to discriminate these clusters. However, 16S rRNA studies have limited use for predicting functional differences or for understanding micro-evolutionary changes in gut communities, as bacteria with almost identical 16S rRNA sequences can exhibit high levels of sequence divergence at other loci and very different gene repertoires [10]–[12]. Consequently, little is known about diversification of bacterial lineages in the gut.

Insect gut communities are relatively small and simple, and thus can serve as model systems to explore diversification in gut bacteria [13]. In particular, honey bees (genus *Apis*) and related bumble bees (genus *Bombus*) harbor characteristic gut communities dominated by <10 bacterial species in three phyla: *Proteobacteria*, *Firmicutes*, and *Actinobacteria*. Most of the bacterial taxa in honey bees are not found in other environments or in solitary bee species but are consistently present and abundant in the guts of adult *Apis* and *Bombus* [14]–[23]. Thus, these bacteria are adapted to live in the guts of social bees and likely possess specific symbiotic mechanisms affecting health of the host. *Apis* and *Bombus* are important pollinators and have suffered from severe population declines [24]–[26]. Therefore, studies on the characteristic bee gut microbiota are of interest not only for basic understanding of microbial communities, but also for potential applications in agriculture and biotechnology.

Metagenomic analyses provided initial insights into the functional gene content of the gut symbionts of the honey bee, *Apis mellifera* [22], and revealed polymorphism within two of the core species, *Gilliamella apicola* (*Gammaproteobacteria*) and *Snodgrassella alvi* (*Betaproteobacteria*), which were each classified as single species on the basis of previous 16S rRNA analyses [20], [21], [27]. Reference genomes of *Apis* and *Bombus* isolates of *G. apicola* and *S. alvi* are now available [28] facilitating comparative analyses of the genomic variation across strains from conspecific and heterospecific hosts.

Here, we used single-cell genomics to investigate the genomic variation in *S. alvi* and *G. apicola* sampled from a single colony of *A. mellifera*. By sorting individual bacterial cells from specific gut regions of adult worker bees, we were able to sequence genomic DNA of four single cells of *S. alvi* and of three single cells of *G. apicola* and compare their genomes against the completely sequenced reference strains. These analyses revealed surprising levels of genomic divergence between strains with near-identical 16S rRNA and illustrate the applicability of single-cell genomics for population genomic studies of host-associated bacteria.

## Results

### Sorting single bacterial cells of *S. alvi* and *G. apicola* from the gut of *A. mellifera*

**Figure 1:**
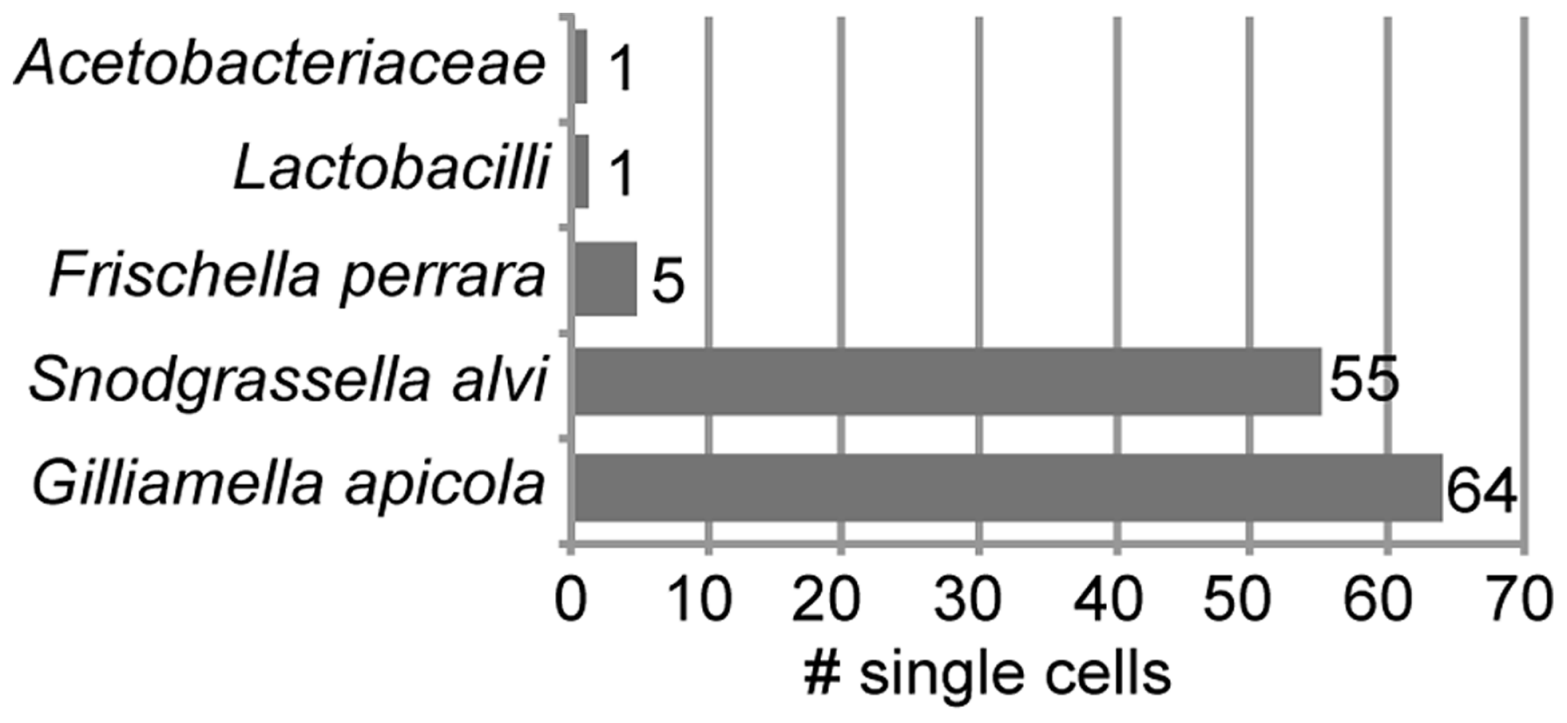
Taxonomic classification of 126 bacterial cells sorted from midguts and ileums of honey bees. Classification is based on best BLASTN hit of partial 16S rRNA sequences. Table S1 provides a complete list of all best BLASTN hits.

We sorted 315 single bacterial cells from homogenate of the midgut and ileum gut regions of ten *A. mellifera* workers collected on the same day from a single colony in West Haven, CT, USA (Figure S1). Following bacterial lysis and multidisplacement amplification (MDA), we obtained detectable DNA enrichment for 300 of the 315 cells (95%) (Figure S2). Of the 315 sorted cells, 216 were confirmed to contain bacterial DNA using PCR of a partial fragment of the 16S rRNA gene (Figure S2). We sequenced 16S rRNA amplicons for a random selection of 126 of these 216 single amplified genomes (SAGs) and found that all 126 belonged to core members of the gut microbiota of *A. mellifera* (Table S1). As expected, most were *S. alvi and G. apicola* (Figure 1). However, a few cells of other core members were also identified, i.e. five cells of *Frischella perrara* (*Gammaproteobacteria*) [29], one *Firmicute*, and one *Alphaproteobacterium*.

### 16S rRNA sequence analysis of *S. alvi* and *G. apicola* SAGs

**Figure 2:**
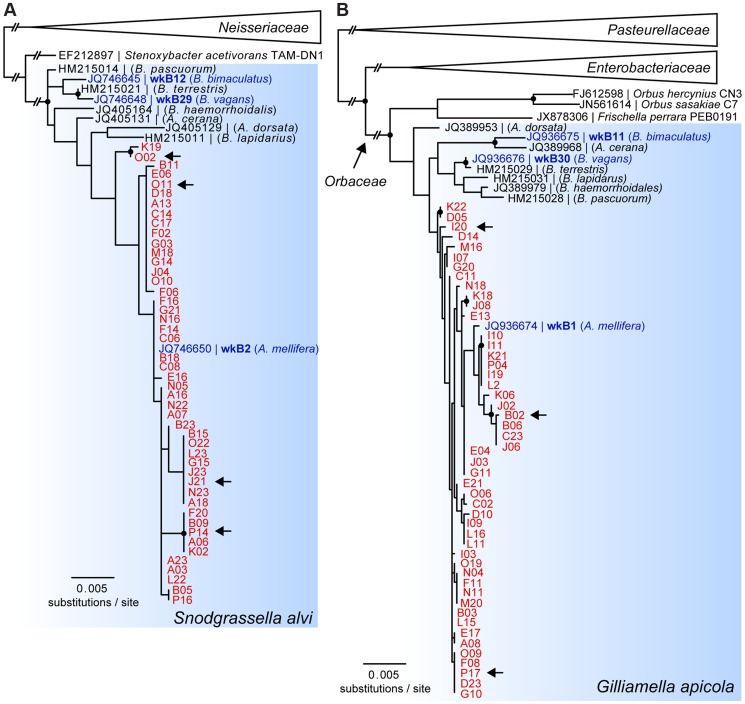
16S rRNA-based maximum likelihood trees of SAGs of (A) *S. alvi* and (B) *G. apicola*. SAGs are highlighted in red and denoted according to their position in the 384-well plate. Reference strains isolated from honey bees and bumble bees with available genome sequences are highlighted in blue. Arrows indicate SAGs selected for genome sequencing. Trees were inferred from (A) 1,229 and (B) 1,167 aligned nucleotide sites of partial 16S rRNA sequences of *S. alvi* and *G. apicola*, respectively. Nodes with bootstrap values ≥80 (100 replicates) are marked with black circles. In (A), *S. alvi* and *Stenoxybacter acetivorans* are part of the larger *Neisseriaceae* family, while in (B) *G. apicola* belongs to the family of *Orbaceae* together with the species *Orbus hercynius*, *Orbus sasakiae*, and *F. perrara*. Branches to distantly related outgroup species are collapsed, and long branches are shortened, as indicated by two interrupting vertical dashes.

Phylogenetic trees based on partial 16S rRNA sequences showed that SAGs of *S. alvi* and *G. apicola* both formed monophyletic clades together with their corresponding type strains, *S. alvi* wkB2 and *G. apicola* wkB1, both previously isolated from *A. mellifera* [27] (Figure 2). Sequences originating from other bee species clustered outside the *A. mellifera*-specific clades, including the *Bombus* isolates for which genome sequences are available (*S. alvi* wkB12 and wkB29 and *G. apicola* wkB11 and wkB30). However, due to the high similarity between 16S rRNA sequences, most nodes within the species-clusters were not supported by bootstrap analyses. The average pairwise sequence divergence (π) between all analyzed SAGs was 0.27% and 0.56% for *S. alvi* and *G. apicola*, respectively. The average π for representative sequences originating from strains of different bee species was higher, at 0.67% and 2.47% for *S. alvi and G. apicola*, respectively.

### Genomic sequencing and *de novo* assembly of *S. alvi and G. apicola* SAGs

To analyze genome-wide diversity, we shotgun-sequenced four SAGs of *S. alvi* (J21, O02, O11, P14) and three SAGs of *G. apicola* (B02, I20, P17) in a single multiplexed Illumina lane (Table 1). SAGs were selected based on distinct positions in the 16S rRNA gene trees (Figure 2) and their low Cp (critical point) values, i.e. the time required to produce half of the maximal fluorescence during the MDA reaction (Figure S2). Low Cp values are indicative for ample template DNA, which should result in a less biased DNA amplification and better coverage of the genome (R. Stepanauskas, unpublished data). For each SAG, we generated first-pass assemblies with SPAdes [30] and detected some low-coverage contigs originating from misassigned reads, i.e., reads assigned to the wrong dataset due to dense clustering on the Illumina flow cell [31] (see Materials and Methods for details). Following removal of misidentified reads, the curated datasets consisting of 25–32 million reads were re-assembled. Resulting contigs were included in subsequent analyses only if they met our quality criteria which were based on read coverage, contig length, redundancy, and homology (see Materials and Methods for details). The final assemblies consist of 259–544 contigs and range in total size from 1.31 Mb to 2.33 Mb (Table 1).

**Table 1:** Genome features of SAGs and comparisons to their reference genome.

						Comparison to reference genome^a^			
SAG sample	Species	Total size (Mb)	Contig count	Contig N50 (bp)	CDS count	% Genome coverage^b^	% 16S rRNA Id^c^	*d*S^d^	*d*N/*d*S^e^
J21	*S. alvi*	2.33	456	44,241	2,414	93	99.68	0.064	0.106
O02	*S. alvi*	1.60	259	37,190	1,622	71	99.29	2.087	0.065
O11	*S. alvi*	1.37	401	27,529	1,620	53	99.54	0.175	0.137
P14	*S. alvi*	1.31	385	22,676	1,498	47	99.48	0.064	0.101
B02	*G. apicola*	1.81	544	14,822	2,062	55	99.15	0.245	0.069
I20	*G. apicola*	2.21	389	43,035	2,377	74	99.02	1.852	0.049
P17	*G. apicola*	1.47	296	31,866	1,611	61	98.95	1.883	0.044

a *S. alvi* SAGs were compared to the reference genome of strain wkB2. *G. apicola* SAGs were compared to the reference genome of strain wkB1.

b Estimated genome coverage is based on the presence of 206 and 189 genes constituting the minimal, essential gene set of wkB1 and wkB2, respectively. The minimal gene sets were determined as described previously [22], [97].

c Id, identities.

d *d*S, rate of synonymous substitutions per synonymous site, averaged over all shared genes.

e *d*N/*d*S, ratio of *d*N (rate of non-synonymous substitutions per non-synonymous site) to *d*S.

### Genomic variation and sequence divergence patterns between SAGs and reference genomes

Based on the coverage of a minimal, essential gene set defined for the fully sequenced reference genomes of *S. alvi* wkB2 and *G. apicola* wkB1, genome completeness of the sequenced SAGs was estimated to range from 47% to 93% (Table 1). We determined orthologous gene sets present in SAGs and reference genomes, mapped these onto the reference genome, and found that large genomic regions were missing from SAG assemblies (Figure S3). Many of these missing regions likely reflect incomplete genome recovery from the single cells. Missing regions are erratically distributed (Figure S3), suggesting that they correspond to random processes (e.g., incomplete single cell lysis and the stochastic nature of single cell MDA) rather than compositional variation among genomic regions, consistent with prior studies [32], [33].

**Figure 3:**
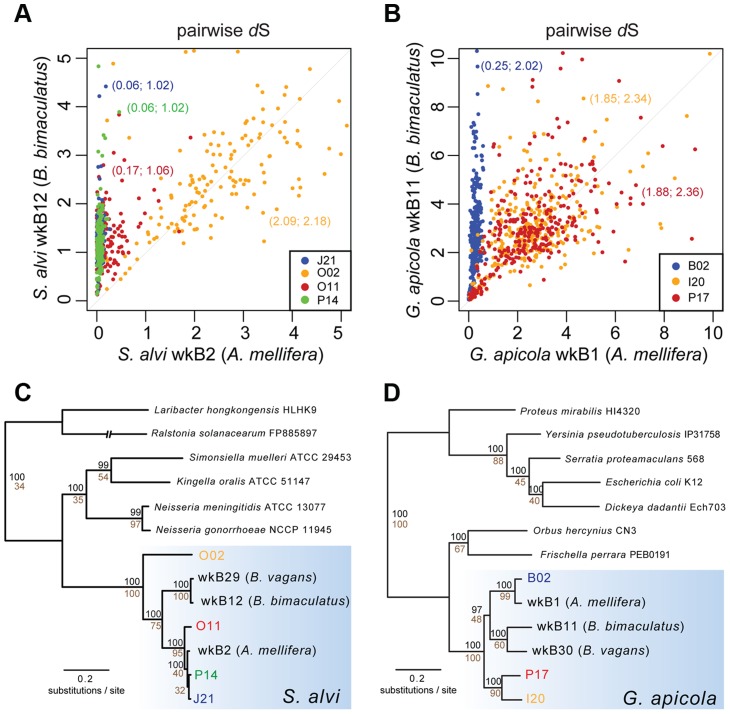
Sequence divergence and phylogenetic analysis of protein-encoding genes of (A and C) *S. alvi* SAGs and (B and D) *G. apicola* SAGs. Pairwise sequence divergence was measured by estimated rates of synonymous substitutions per site (*d*S) for (A) 226 orthologs of *S. alvi* and (B) 348 orthologs of *G. apicola*. Pairwise *d*S values of SAGs and reference genomes of *A. mellifera* isolates (in (A), *S. alvi* wkB2; in (B), *G. apicola* wkB1) are plotted on the x-axes. Pairwise *d*S values of SAGs and reference genomes of bumble bee (*Bombus bimaculatus*) isolates (in (A), *S. alvi* wkB12; in (B), *G. apicola* wkB11) are plotted on the y-axes. Mean *d*S values are given in parentheses (SAG compared to *A. mellifera* isolate; SAG compared to *B. bimaculatus* isolate). For visualization purposes, genes with unrealistically high *d*S values were excluded from representation. Complete data is presented in Figure S5. Note that genes with *d*S value ≥3 can be considered at or near saturation due to the four possible bases in the genetic code. (C and D) Maximum likelihood trees based on the concatenated alignments of 114 and 211 conserved orthologs of *S. alvi* and *G. apicola*, respectively. Values above branches represent bootstrap values ≥80 for 100 replicates. Values below branches indicate the percentage of single-gene trees with congruent topology at this node.

Despite missing regions, we determined a shared set of 239 genes for *S. alvi* and 400 genes for *G. apicola* (Figure S4). Analysis of these orthologs revealed extended intraspecific variation in divergence at synonymous sites (*d*S, estimated rate of synonymous substitutions per site). This was unexpected, because the analyzed strains show similar divergence at 16S rRNA gene loci (Table 1). Genome-wide *d*S values between SAGs and reference genome range from 0.064 to 2.087 for *S. alvi* and from 0.245 to 1.883 for *G. apicola* (Table 1). *d*S values of individual genes show similarly extreme variation in divergence across strains (Figures 3A and 3B). With many orthologs exhibiting *d*S values near or at saturation (i.e. *d*S values ≥3), O02 of *S. alvi* and I20 and P17 of *G. apicola* are the most divergent SAGs compared to their respective reference genome. These SAGs seem to be almost as divergent from other honey bee isolates as they are from strains isolated from *Bombus* species (Figures 3A and 3B).

For both *S. alvi* and *G. apicola*, phylogenetic trees inferred from concatenated protein-encoding genes differ from 16S rRNA gene trees (Figures 3C and 3D). Strains from *A. mellifera* do not form a single monophyletic clade exclusive of *Bombus* strains. For *S. alvi*, three of the four SAGs (J21, O11, P14) and the reference strain from *A. mellifera* (wkB2) form a tight clade, but the most divergent SAG, O02, occupies a basal branch within the *S. alvi* cluster. For *G. apicola*, two strains from *A. mellifera* (B02 and wkB1) form a clade that is sister to the clade of *Bombus* strains (wkB11 and wkB30); the other *G. apicola* strains from *A. mellifera* (P17 and I20) form a more basally branching clade. Thus, trees for both *G. apicola* and *S. alvi* support divergence among *A. mellifera* strains that started before and continued after the divergence from *Bombus* strains. Most relevant branches are supported by high bootstrap values (≥80) and by topology concordance of the majority of the single gene trees (≥50% of the analyzed genes) (Figures 3C and 3D).

### Patterns of recombination between SAGs and reference genomes

For both *S. alvi and G. apicola*, 16S rRNA sequences are highly similar across closely related and highly divergent strains. This could reflect the occurrence of frequent homologous recombination at 16S rRNA loci resulting in sequence homogenization [34]. Using the four-gamete test under the infinite sites assumption (i.e. repeat mutations have zero probability), we found that at least 1 and at least 8 recombination events must have occurred between the 16S rRNA sequences of the ancestors of SAGs of *S. alvi* and *G. apicola*, respectively. This finding is concordant with a previous study showing homologous recombination of 16S rRNA genes in both *S. alvi* and *G. apicola*, with higher rates in *G. apicola* [20]. To test whether other genes of *S. alvi* and *G. apicola* show signs of recombination, we (i) examined single gene trees for topology discordance with the concatenated gene tree, (ii) analyzed patterns of sequence divergence at synonymous sites, (iii) determined the frequency at which substitutions occurred by mutation or recombination, and (iv) detected intragenic recombination events between orthologous genes.

### Patterns of recombination in *S. alvi*

The O02 lineage of *S. alvi* appears to have low recombination rates with the other analyzed strains, as most single gene trees (75%) support its basal position (Figure 3C), and most trees with incongruent topologies have weak support for the position of O02 (Figure S6). In contrast, for the more closely related strains (J21, P14, O11, and wkB2), many single gene trees show discordant topologies (Figure S6), indicating either recombination and/or insufficient phylogenetic signal.

**Figure 4:**
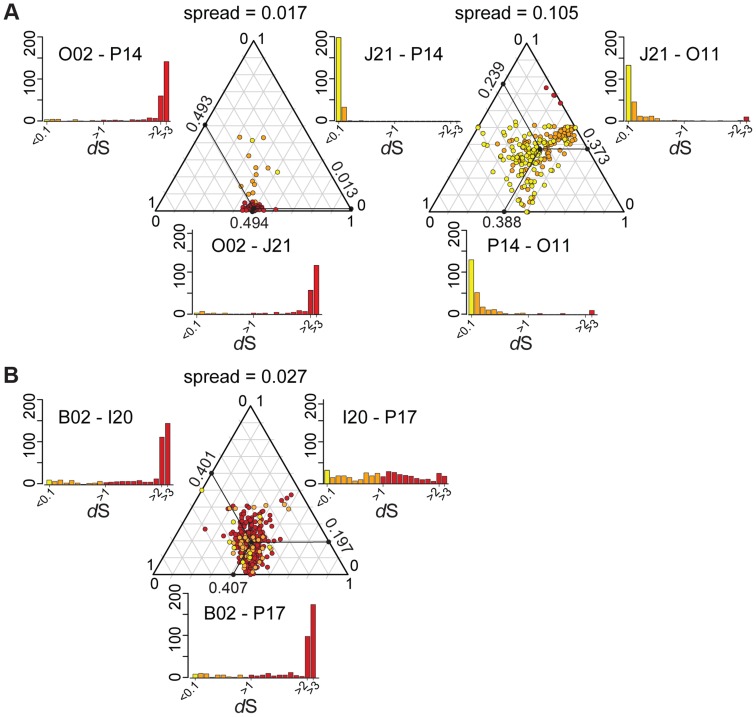
Ternary plots of sequence divergence at synonymous sites. Plots show gene-to-gene variation for synonymous substitution frequencies, for (A) 239 orthologs of *S. alvi* SAGs and (B) 400 orthologs of *G. apicola* SAGs. Each dot represents a triplet of orthologs. The sum of all three pairwise *d*S values have been normalized to 1 and plotted onto the ternary plot. The mean relative *d*S value for each pair is shown on the axes of the ternary plot. Distributions of absolute *d*S values are shown in histograms for each pair next to the ternary plot. X-axes show *d*S value categories, y-axis show number of genes. Colors represent the maximum absolute *d*S value in each comparison, with yellow for *d*S<0.1, orange for *d*S≥0.1, and red for *d*S≥1. Spread of each ternary plot is the median distance to the average point. *d*S values have been restricted to a maximum of three, because higher values cannot be reliably estimated and suggests substitution frequencies to be at saturation. For *S. alvi*, two ternary plots are shown to present comparisons among all four SAGs.

**Figure 5:**
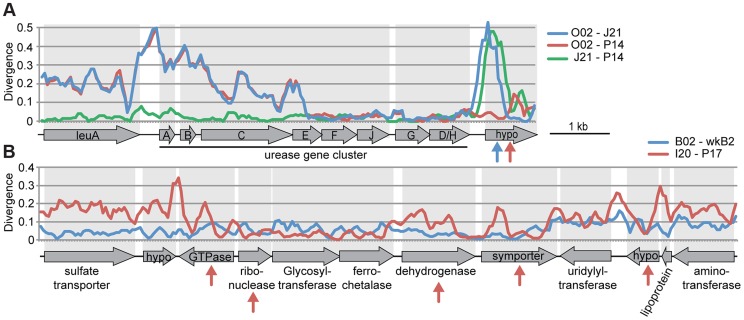
Recombination within genomic regions of (A) *S. alvi* and (B) *G. apicola*. Sequence divergence at all sites is plotted for pairwise comparisons in a sliding window of 200 nucleotides with a step size of 50 nucleotides. Arrows indicate genes for which intragenic recombination between pairs has been detected with the program Geneconv. Arrow colors correspond to the different pairwise comparisons. (A) Sequence divergence over the urease gene cluster of *S. alvi*. Note the drastic decrease in sequence divergence between O02 and the other two SAGs in urease gene E. Recombination seems also to have occurred in the gene encoding a hypothetical protein (hypo) at the end of the gene cluster. (B) Sequence divergence over a genomic region of *G. apicola*. I20 and P17 show variation in sequence divergence, particularly in genes, for which intragenic recombination was detected. In contrast, no evidence for recombination could be found between B02 and wkB2.

In the absence of recombination, *d*S values for a pair of genomes will reflect their divergence time and will show a consistent pattern across genes, but recombination will cause some genes to have anomalous *d*S values. We plotted *d*S values in ternary plots, in which the sum of all values for three pairwise comparisons equals 1. For the ternary plot of O02, P14, and J21, most genes exhibit similar divergence patterns, with *d*S values ∼40× lower for J21-P14 than for J21-002 or P14-O02 (Figure 4A), indicating deeper branching of 002 and little subsequent recombination. Of the 13 genes dispersed over the plot area. 12 fell within three regions in the wkB1 reference genome, one encoding genes for urease and two encoding genes for efflux permeases (Table S2). A sliding window analysis over the aligned sequences of the urease-encoding gene cluster showed that sequence divergence between O02 and both J21 and P14 drops from high to very low (Figure 5A), suggesting that the recombination breakpoint is located in the middle of the locus. Ternary plot analysis of the three closely related SAGs, J21, O11, and P14, showed much more dispersal of genes (Figure 4A), mostly reflecting low *d*S values that do not differ significantly. However, a considerable number of the dispersed genes show *d*S values >0.1 (Figure 4A), suggesting that intraspecific recombination contributed to this variation in sequence divergence.

To determine the relative importance of recombination versus mutation in sequences of *S. alvi* strains, we estimated the ratio (r/m) at which substitutions are generated via recombination (r) or mutation (m) across the 239 shared genes. Most r/m values were <1 for the closely related *S. alvi* strains, suggesting that mutations contribute more to their evolution. For the distant strain, O02, no reliable estimates could be obtained due to saturation of substitutions at most sites (Table S3).

Small fragments of genes exchanged by recombination might be missed by our phylogenetic or divergence analyses based on whole genes. Therefore, we tested for the occurrence of intragenic recombination within shared genes and found that 7–19% of genes carried signs of past recombination events (Figure S7). In agreement with its divergent phylogenetic position, O02 had the fewest genes with intragenic recombination (7%), while all other strains had at least 15% affected genes. Average recombination fragment length generally decreased with increasing phylogenetic distance of the analyzed strains (Figure S7).

### Patterns of recombination in *G. apicola*

Most genes display congruent topologies for the splits between the three SAGs and the reference strain of *G. apicola*, providing no evidence for frequent recombination among their ancestors (Figure 3D). Consistent with this, the ternary plot analysis does not detect much variation in relative *d*S among the shared genes, as indicated by low dispersal over the plot area (Figure 4B). However, *d*S is near saturation for many genes, possibly obscuring evolutionary rate differences. Nevertheless, a slight dispersal of *d*S values along the axis plotting the comparison of I20 and P17 is evident. Concordantly, *d*S values for these two strains vary markedly among orthologs, in contrast to the other pairwise comparisons, for which most orthologs exhibit uniform *d*S values (Figure 4B, Figure S8). I20 and P17 form one of the two *A. mellifera*-associated clades of *G. apicola*, and the variation in *d*S suggests a high frequency of recombination in this particular sub-lineage. This was confirmed by estimates of r/m, revealing very high rates of recombination (5.1–23.5) for I20 and P17. In contrast, r/m ratios for B02 and the reference genome wkB1 are much lower (0.4–0.8) (Table S3). The differences in recombination frequencies among *G. apicola* strains are further corroborated by the analysis for intragenic recombination. Recombination is evident in all pairwise comparisons, but is highest for I20 versus P17 (Figure S7B) for which 15% of shared genes (60 of 400 genes) show evidence of at least one recombination event. In comparison, only 2–4% of the 400 shared genes show signs of recombination between any other pair of *G. apicola* genomes. A sliding window analysis over a genomic region of *G. apicola* illustrates these differences in recombination frequency between P17 and I20, and B02 and wkB2 (Figure 5B).

### Functional differences between strains of *S. alvi* and *G. apicola*

**Figure 6:**
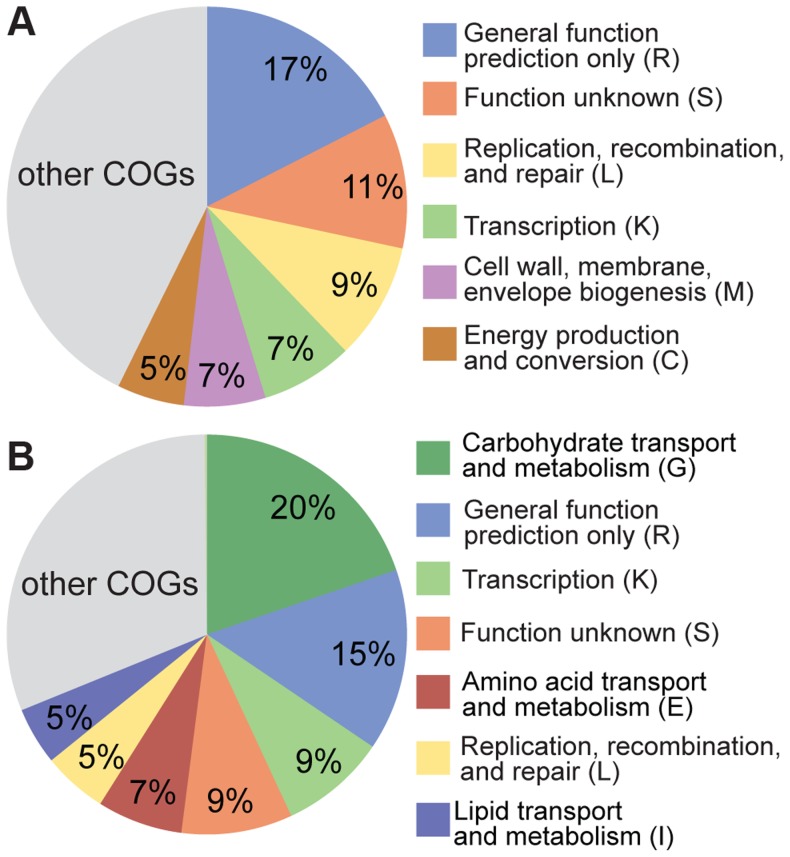
Functional classification of the accessory genes of (A) S. *alvi* SAGs and (B) *G. apicola* SAGs based on COG categories. 303 of 755 accessory genes of *S. alvi* SAGs and 476 of 851 accessory genes of *G. apicola* SAGs could be classified into COGs. Categories covering ≥5% of the classified accessory genes are shown. Minor categories (<5%) are summarized in the grey colored area of the pie charts. Categories R, S, and L include genes associated with mobile genetic elements.

Despite the clear evidence for recombination in both *S. alvi and G. apicola*, strains from the same bee colony can be highly divergent, potentially reflecting adaptation to distinct ecological niches in the bee gut. To test for differences in functional gene content between strains, we determined the accessory gene pool of SAGs, which we defined as the genes present in SAGs but absent from the completely sequenced reference genomes. Based on this criterion, we found 755 and 851 accessory genes for *S. alvi* and *G. apicola*, respectively (Figure S4). For *S. alvi*, the accessory gene pool is dominated by categories covering a broad range of functions (Figure 6A). Among others, we found a considerable number of genes associated with mobile elements such as phages, plasmids, or transposons, and restriction-modification systems. In agreement with its distant phylogenetic position, strain O02 has the largest accessory gene pool, with 258 unique genes (Figure S4), suggesting that O02 differs substantially from other sampled strains in its functional capabilities. However, many of these genes are annotated as hypotheticals, preventing prediction of their functional roles.

For *G. apicola*, 20% of the accessory genes encode carbohydrate-related functions, including many transporters of the phosphotransferase system and major facilitator families, and another 7% corresponds to amino acid transport and metabolism (Figure 6B). These marked differences in gene content linked to metabolic functions suggest distinct ecological roles and effects in the host.

## Discussion

Studies of bacterial diversity typically focus on 16S rRNA gene sequences. But such analyses give only limited understanding of bacterial diversification. We found high variation in intraspecific sequence divergence for both *G. apicola* and *S. alvi*, despite uniformly high 16S rRNA similarity. Some strains originating from *A. mellifera* are as divergent from each other as from strains isolated from *Bombus* species. They exhibit saturation of *d*S and form deep-branching lineages in phylogenies based on protein-encoding genes. Despite high sequence divergence, interstrain recombination was evident, but its frequency varies and generally decreases with increasing divergence between strains. The accessory gene sets of *G. apicola* imply that strains differ in metabolic functions, which could reflect divergent adaptation to different niches in the gut environment.

### Application of single-cell sequencing for population genomic analyses

We sorted single cells directly from their environment to obtain an unbiased picture of genomic variation within populations. While isolates of *G. apicola* and *S. alvi* have been grown in the laboratory [27], culturing often introduces sampling biases [35]–[38], as certain strains possess metabolically costly genes or lytic phages which hinder growth in culture [39]. The *A. mellifera* gut microbiota is particularly suitable, because its low species richness facilitates high-frequency retrieval of single cells of the same species (i.e., cells with near-identical 16S rRNA sequences). By only targeting the bee gut ileum and midgut, we could increase the sorting frequency of *G. apicola* and *S. alvi*, which dominate in these regions [40]. Single-cell enrichment of specific bacteria from more complex communities, such as those in mammalian guts, may require a higher sorting throughput or specific labeling with fluorescent probes.

An obvious limitation of single-cell genomics for population genetic analysis is the incomplete recovery of genomes from single cells [41], [42]. We obtained 239 and 400 shared genes with an average genome completeness of 66% and 63% for four *S. alvi* SAGs and three *G. apicola* SAGs, respectively. These estimated genome recoveries were in the upper range of previous single-cell studies [35], [38], [43]–[47] and provided abundant genomic information for our analyses. Nevertheless, it is important to note that the number of shared genes rapidly decreases as SAGs are added to the analysis, due to the higher likelihood of a given gene being absent from one of the samples. Population genetic studies of larger SAG datasets would therefore require higher average genome coverage and new computational tools to take full advantage of partial genomes. Recent studies have shown that partial genomes can also be obtained from metagenomic datasets [48]–[51]. While metagenomic approaches might be cost-effective, reconstruction of closely related genomes is difficult, hindering evolutionary analysis of bacterial populations.

### Do divergent strains of *S. alvi* and *G. apicola* correspond to separate species?

There is no generally accepted species concept for bacteria, and microbiologists use different criteria to delineate species [52]–[54]. A pragmatic and commonly applied convention uses 16S rRNA sequence similarity to define OTUs, with an arbitrary cut-off of 97% for species delineation [55]. However, this criterion is not an indicator of biologically meaningful boundaries between ecologically and genetically distinct populations, and bacterial strains with near-identical 16S rRNA may be adapted to different ecological niches or harbor distinct functional capabilities [11], [12], [35], [56], [57]. Most *G. apicola* and *S. alvi* strains investigated in this study share 99–100% sequence identity in 16S rRNA with their respective type strain (Table S1), but often show much higher divergence at other loci as well as very different gene repertoires (Figure 3 and Figure S4). This appears to reflect the slow evolution of rRNA genes. But compared to other pairwise analyses of bacteria [58], the extent of divergence of protein-encoding genes relative to 16S rRNA divergence is exceptionally high for the two honey bee gut symbionts analyzed in this study. Concerted evolution or ongoing homologous recombination at 16S rRNA loci (even when other genomic regions continuously diverge) could be two possible explanations for this phenomenon. It is not apparent why gut bacteria would have stronger purifying selection on rRNA genes than any other bacteria resulting in concerted evolution of these loci. Further, our analyses provide evidence that sequence homogenization likely originates from recombination. First, we found incongruence between tree topologies for 16S rRNA and those for protein-encoding genes (Figure 2 and Figure 3). Second, we detected recombination breakpoints in 16S rRNA sequences, which was consistent with a previously published analysis [20]. Consequently, 16S rRNA sequences fail to portray the extensive genetic diversity present in *S. alvi* and *G. apicola* populations, and other genomic regions must be considered to demarcate divergent intraspecific lineages. For example, O02 of *S. alvi* appears to have irreversibly separated from other strains. While frequent recombination and genome cohesion was evident among other *S. alvi* strains, O02 has undergone recombination in only a few genomic regions (Table S2). These few genes could correspond to adaptive functions important for survival in the shared habitat. The urease gene cluster, for example, might be responsible for tolerance to acidic conditions in the *A. mellifera* gut, based on the role of this enzyme in other host-associated bacteria [59].

Notably, divergent strains within *S. alvi* and *G. apicola* co-exist in an individual bee [20], suggesting that they may occupy different niches and constitute distinct ecotypes [60]. In *G. apicola*, a large proportion of the accessory gene pool consists of carbohydrate-related functions (Figure 6), which might play a role in divergent adaptation to different metabolic niches. This corroborates previous results showing that strains of *G. apicola* differ in ability to breakdown pectin, a major component of the cell wall of pollen [22], the major source of dietary protein of honey bees. Furthermore, the fully sequenced honey bee-associated strain wkB1 has a larger genome (3.14 Mb) than the two *Bombus-*associated strains (2.26 Mb, 2.32 Mb), largely due to an expanded set of genes involved in carbohydrate metabolism [28]. These results suggest the possibility that *G. apicola* strains affect the use of diverse carbohydrates present in the diets of different honey bee colonies.

### Does intraspecific diversity influence host health?

Social bees are key pollinator species in almost all terrestrial ecosystems, including agricultural systems. In recent years, *A. mellifera* has undergone colony losses [25], and *Bombus* species have also suffered from population declines and extinctions [24], potentially influenced by pesticide usage and interactions with parasites. No consistent changes in microbiota are apparent in failing *A. mellifera* colonies [21], [61], but studies have been based on 16S rRNA, which lacks resolution to reveal differences in strain composition. Strain composition in the gut could affect nutrient availability or resistance to parasites. Preliminary support for such effects comes from experiments showing differences in *G. apicola* strains for pectin catabolism [22] and from experiments on *Bombus* showing that particular sources of gut symbionts vary in levels of protection against protozoan parasites [62]. Moreover, strains might vary in overall effects on hosts, from beneficial to neutral or even detrimental. All sampled *A. mellifera* workers harbor *G. apicola* and *S. alvi*, but differ in strain composition [20], and these differences potentially impact health of bee colonies.

### What has caused the divergent evolution of *S. alvi* and *G. apicola*?

Diversification of *S. alvi* and *G. apicola* likely occurred within the bee gut environment, as both species have been detected exclusively in the guts of *Bombus* and *Apis* [14], [16]. It is possible that they descend from ancestors that colonized an *Apis-Bombus* ancestor living ∼85 million years ago [63]. Divergence of strains in different *Apis* and *Bombus* species reflects host evolutionary relationships, at least in part [64]. When transmission is largely intraspecific, divergence of strains confined to different host species is expected, and is likely reinforced by symbiont-host coevolution, resulting in barriers to colonization of non-native hosts. Parallel cases are *Xenorhabdus* species specialized to particular species of *Steinernema* nematodes [65] and *Lactobacillus reuteri* strains adapted to different vertebrate hosts [66], [67].

Our study focuses on strain variation that appears to have arisen due to diversification within a single host species, *A. mellifera*; this situation likely parallels that of the human gut microbiota [3], [68]. Diversification is likely driven by divergent selection reflecting ecologically distinct niches in the gut, but such diversifying selection might be countered by recombination with homologous DNA from other strains. Temporary isolation of host populations and colonies, followed by recontact and exchange of symbionts, might generate ecological and genomic diversity among symbiont strains. In *A. mellifera*, symbiont exchange among colonies likely occurs occasionally via robbing behaviors or foraging at the same flowers. The mode of colony founding, by a swarm containing hundreds of workers and a queen, also favors maintenance of strain diversity, because it does not impose a bottleneck in numbers of gut bacteria. In contrast, *Bombus* colonies are initiated annually from a single queen bee, potentially imposing a bottleneck that reduces diversity of gut bacteria. However, whether strain diversity of *S. alvi* and *G. apicola* is lower in *Bombus* hosts is unknown.

### How general are our findings about the microevolution of gut bacteria?

The bacterial diversity in *A. mellifera* gut ecosystems consists of many closely related taxa, but relatively few deep-branching lineages, a pattern similar to that in mammalian gut microbiota [3], [5], [69]. Another parallel with mammalian systems is that strains categorized as single species on the basis of 16S rRNA can have extensive differences in genome content: as for *S. alvi* and *G. apicola*, over 25% of each genome may be unalignable across strains of human gut bacterial species [70]. In the human gut, strains are persistent within individual hosts and tend to be shared among relatives living together [70], [71]. Colony-specific strain composition also appears to occur in *A. mellifera* [20].

Although 16S rRNA sequences are typical markers for assessing diversity in bacterial communities, we found that they correlate poorly with genomic content and divergence at protein-coding loci. Because most studies of genome-wide patterns of variation are based on metagenomic samples which do not reveal linkage of 16S rRNA and protein-coding genes (e.g., [71]), it is unclear how often this discrepancy occurs. We propose the following model for how this might evolve. If populations are isolated, for example in different bee colonies, then their genomes will begin to diverge. However, protein-coding genes, particularly synonymous sites, will diverge faster than rRNA genes, in which contiguous regions are conserved due to strong purifying selection to maintain function. If recontact of populations occurs following an appropriate time interval, regions of the rRNA may retain sufficient similarity to recombine through homologous recombination pathways, which require near-identity for a region of >50 base pairs [72], while many or all protein-coding regions may exceed this divergence threshold. In this sense, the rRNA genes have not yet “speciated”, while protein-coding regions have. Ongoing coexistence could result in extensive recombination and homogenization at rRNA loci and continued divergence of protein-coding loci, increasing the discrepancy between their divergence levels. The continued coexistence of strains also suggests ecological specialization maintaining strain variation, as proposed for other communities (e.g., [73], [74]). Such specialization would reinforce the highly distinct gene repertoires of strains, such as those we observed. Experiments on metabolism and host-relationships of isolates will illuminate this possibility and reveal the extent to which strain divergence and ecological differentiation correlate.

## Materials and Methods

### Single-cell sorting of bacteria from *A. mellifera* guts

10 worker bees were collected from inside a single hive in West Haven, CT, USA. The midgut and the ileum (anterior part of the hindgut) were dissected with sterile forceps and homogenized with a pestle in 6% betaine in 1× PBS. The homogenate was pipetted into a fresh tube avoiding gut tissue debris and frozen at −80**°**C. Aliquots were shipped on dry ice to the Single Cell Genomics Center at the Bigelow laboratory, Maine, USA, for fluorescence-activated cell sorting (FACS), single-cell lysis, and multiple displacement amplification (MDA) following procedures described previously [75].

### Genotyping of single cells

An initial qPCR screen for the 16S rRNA gene was performed with primers 27F (AGR GTT YGA TYM TGG CTC AG) and 907R (CCG TCA ATT CMT TTR AGT TT) on each single-cell sample in the 384-well plate. None of the negative control samples gave a positive PCR signal (Figure S2). Initially, amplicons from 94 SAGs were selected for Sanger sequencing with primer 907R. For phylogenetic analyses, we generated longer 16S rRNA gene PCR amplicons with primers 27F and 1507R (TAC CTT GTT ACG ACT TCA CCC CAG). Amplicons were Sanger-sequenced and assembled into near-full length 16S rRNA gene sequences. A total of 126 SAGs were genotyped by using the partial 16S rRNA sequences as queries in BLASTN against the NCBI non-redundant database and against the 16S rRNA gene sequences of *F. perrara* PEB0191 [29], *S. alvi* wkB2, and *G. apicola* wkB1 [27] (Table S1).

### Single-cell genome sequencing, assembly, and annotation

Selected MDA products were sequenced on an Illumina HiSeq 2000 machine at the Yale Center for Genome Analysis. Illumina paired-end libraries with approximate insert sizes of 400 bp were constructed following Illumina standard protocols for genome sequencing using four PCR amplification cycles with the Bio HiFi polymerase (Kapa Biosystems, Woburn, MA, USA). These libraries were sequenced as part of a larger multiplexed pool in a single 2×76 bp lane. Sequencing reads were corrected with BayesHammer and first-pass assemblies generated with SPAdes using standard parameters [30], [76]. Illumina's multiplexing technology has a relatively high error rate (0.3%) for assigning reads to the correct library adapter sequence [31]. The higher the read coverage for a given region, the more reads of this region are being misassigned. Sequencing data obtained from single-cell derived MDA products typically reveal large variation in read coverage [76], [77], with some regions being covered by >10,000×. We determined that a substantial number of reads were misassigned and assembled into contigs of low coverage (mostly <10×), if the read coverage of a particular region in the original dataset exceeded 5,000× to 10,000×. To identify and remove such misassigned reads, we mapped every Illumina read dataset against assembled regions of other datasets exceeding a read coverage of 5000×. Reads were mapped with SOAP2 v2.21 [78] allowing for two mismatches per read. Reads that mapped with an average read coverage of ≤20× over the length of the read were removed from the dataset. Reads that mapped with an average read coverage of >20× were searched with BLASTN against the other datasets to avoid removing correctly assigned reads from highly conserved regions. Cleaned read datasets were again corrected with BayesHammer and assembled with SPAdes [30], [76]. The resulting assemblies were annotated with the IMG/MER system (Integrated Microbial Genomes and Metagenome Expert Review system) using the standard metagenome pipeline [79]. To remove sequences originating from potential DNA contamination during cell sorting or MDA reaction, or from spontaneous DNA synthesis, we excluded contigs fulfilling any of the following three criteria: (i) contig length <250 bp, (ii) contig length <500 bp, and read coverage <5× or no BLASTX hit to the reference genomes wkB1 and wkB2 (E-value cutoff of 10^−5^), (iii) contigs with no BLASTX hit to any bacterial sequence in the non-redundant database. We also removed contigs identical to larger contigs in the same assembly, because these redundant contigs typically present assembly artifacts due to the high read coverage of certain regions.

### Ortholog analysis

Orthologous gene sets were determined with OrthoMCL [80], for *S. alvi* SAGs J21, O02, O11, P14 and the reference genomes wkB2 (CP007446), wkB12 (JFZW00000000), wkB29 (JFZV00000000), and for *G. apicola* SAGs B02, I20, P17 and reference genomes wkB1 (CP007445), wkB11 (JFON00000000), wkB30 (JFZX00000000). To this end, separate all-against-all BLASTP searches with the protein sequences of *S. alvi* genomes and *G. apicola* genomes were performed. We only considered BLASTP hits with ≥50% protein identity, covering >50% of both query and hit protein length. Based on these BLASTP hits, CDSs were clustered into sets of homologs using the MCL algorithm [80]. Ortholog clusters of SAGs and reference genomes from *A. mellifera* were extracted and visualized as Venn diagrams (Figure S4). Paralogs were identified within these clusters and the paralog copy with the highest similarity to the other sequences was retained in the cluster. CDSs not belonging to any homolog cluster were classified as remnants, if they had a partial BLASTP hit in any other genome of the same species (alignment length <50% over the length of the hit). They were classified as genome-specific genes, if they had no BLASTP hit in the other genomes of the same species (E-value cutoff of 10^−5^).

### Phylogenetic analysis

16S rRNA sequences were aligned with ClustalW [81] and overhanging ends removed. Phylogenies were inferred with PhyML [82] as implemented in Geneious R6 (http://www.geneious.com/) using the GTR model with substitution rate categories set to four and all other parameters being estimated. Phylogenetic analyses of protein-encoding genes of *S. alvi* and *G. apicola* strains were conducted for genes having an ortholog in all outgroup species. These orthologs were identified with OrthoMCL comparing the reference genome of wkB2 (*S. alvi*) and wkB1 (*G. apicola*) and the complete genomes of six betaproteobacterial species and seven gammaproteobacterial species, respectively (see Figure 3A and 3B). We applied the same BLASTP hit cutoffs as before. A total of 114 and 211 genes for *S. alvi* and *G. apicola*, respectively, were found to have an ortholog in all ingroup and outgroup genomes. These genes were aligned on protein sequence level with MUSCLE [83] and back-translated into aligned DNA sequences with a custom-made Perl script. Single gene trees were inferred with Garli 2.0 [84] using the model of evolution predicted by jModelTest 2 for each gene [85]. To infer the multilocus sequence phylogenies, DNA alignments were concatenated and maximum likelihood phylogenies inferred with Garli 2.0. 100 non-parametric bootstrap trees were calculated for the concatenated alignments and the resulting supports for each split mapped with SumTrees [86] onto the maximum likelihood trees. To summarize the number of single gene trees supporting each split of the multilocus sequence phylogenies, we used the commands ‘Constraints’ and ‘Filter’ in PAUP 4.0 [87].

### Analysis of sequence divergence

Nucleotide diversity (π) of 16S rRNA sequences within *S. alvi* and *G. apicola* was calculated with DNAsp v5 [88]. Pairwise sequence identity between 16S rRNA sequences of SAGs and reference genomes were obtained with ClustalW as implemented in Geneious R6. To estimate the average pairwise sequence divergence at synonymous sites between orthologs of sequenced SAGs and reference genomes, orthologs were aligned as described before. Pairwise sequence divergence was based on maximum likelihood estimation of the synonymous substitution frequency per site (*d*S) using the program codeml implemented in PAML 4.7 (runmode = −2, CodonFreq = 2) [89]. We obtained mean pairwise *d*S and *d*N/*d*S values between SAGs and reference genomes by running codeml on the concatenated alignments of all shared genes (226 genes for *S. alvi* and 348 genes for *G. apicola*, including genomes of *Bombus* strains). Ternary plot analyses were conducted on genes shared between all *A. mellifera* strains (239 genes for *S. alvi* and 400 genes for *G. apicola*), following previously published methods [90]–[92]. In short, relative levels of *d*S values between ortholog triplets of SAGs were calculated and plotted with R using the ‘triangle.plot’ function [93]. The spread of the data points was calculated by averaging the distances between normalized *d*S values of each ortholog to the mean normalized *d*S value.

### Detection of recombination

The minimum number of recombination events in 16S rRNA gene alignments was calculated using the four-gamete test implemented in DNAsp v5 [88]. Sliding window analyses of nucleotide divergence over genomic regions were calculated with DNAsp v5 using the function ‘Polymorphism and Divergence’ with the Jukes-Cantor correction. For this analysis, genomic regions were aligned with ClustalW as implemented in Geneious R6 and stripped from all alignment gaps. To calculate the r/m ratios, two independent runs with the program ClonalFrame [94] were performed on orthologs shared between SAGs and reference genomes from *A. mellifera*. Each run consisted of 100,000 iterations, with a burn-in of 50,000 iterations. Parameters were recorded every 100^th^ iteration. The r/m values were calculated from the output data of the two separate runs using two different methods. The first method considered all positions in the data, independent of the probability of a substitution at each site [95]. The second method only considered positions where the probability of a substitution by either mutation or recombination was ≥0.95 [90]. The program Geneconv was used to detect intragenic recombination events [96]. Different mismatch penalties (gscale = 0, 1, or 2) were used to identify recombination events of different ages. We only considered global inner (GI) fragments, i.e. sequences that result from recombination of other sequences in the alignment. We applied a Karlin-Altschul p-value cutoff of 0.05. The average fragment length for each pairwise comparison was calculated from all significant GI fragments.

### Data deposition

The sequences of SAGs B02, J21, I20, O02, O11, P14, and P17 are deposited in Genbank under accession numbers JAIM00000000, AVQL00000000, JAIN00000000, JAIL00000000, JAIK00000000, JACG00000000, and JAIO00000000.

## Supporting Information

Figure S1 Flow cytometric dot plot of honey bee gut homogenate labeled with SYTO 9 for DNA. A combination of regions R2 and R3 were employed to separate bacterial cells from other particles.
(TIF)

Figure S2 Reaction kinetics summary of real-time multiple displacement amplification (rtMDA) and quantitative PCR (qPCR) of the 16S rRNA gene for single cells sorted into a 384-well microplate. “A” indicates wells with no cells deposited (negative controls); “B” indicates wells (315 in total) with individual cells; and “C” indicates wells with 10 cells (positive controls). Well colors indicate real-time PCR critical point (Cp) values, i.e. the time required to produce half of the maximal fluorescence of the SYTO9 DNA stain during the rtMDA reaction. Green circle colors in wells indicate positive qPCR reactions with Ct values (numbers in circle) significantly lower than Ct values of negative controls. Single cells of *S. alvi* and *G. apicola* selected for whole-genome sequencing are highlighted with green and blue colored frames, respectively.
(TIF)

Figure S3 Mapping of orthologous genes of SAGs onto the reference genome of (A) *S. alvi* wkB2 and (B) *G. apicola* wkB1. Starting from outside, the first circle shows the scale of the reference genome representation in grey- and white-colored steps of 100 kb. The second and third circles (green color) depict the genes on the plus and minus strand of the reference genome. The blue circles represent genes of each SAG for which an ortholog has been identified in the reference genome. The blue color range denotes protein identity between SAG and reference genome according to the scale next to the genome circle. Note the differences in protein identities between different SAGs and reference genome reflecting the high variation in sequence divergence within *S. alvi* and *G. apicola*. Numbers in parentheses denote genome-specific genes neither shared with the reference nor with any other sequenced SAG of the same species (see also Figure S4).
(TIF)

Figure S4 Venn diagrams showing number of orthologs between SAGs and reference genome for (A) *S. alvi* and (B) *G. apicola*. Remnants (r, genes which have partial hits to other genes), paralogs (p), and small genes (<50 aa, potential false positives) were subtracted from the number of genome-specific genes.
(TIF)

Figure S5 Plots show sequence divergence at synonymous sites (*d*S) of core genes for (A) *S. alvi* SAGs and (B) *G. apicola* SAGs (as shown in Figure 3, but on larger scales). Genes with *d*S values ≥3 can be considered at saturation due to the four possible bases in the genetic code. Inset shows all genes in one plot including those with unrealistically high *d*S values.
(TIF)

Figure S6 Summary of single gene tree topologies. Possible topologies are indicated by quartet representations. Values present the number of trees congruent with the depicted topology at the node indicated by an asterisk, divided into trees with and without Bootstrap (Btrps) support of ≥80. Relative values are also given (%). Data was extracted from 114 and 211 single gene trees of *S. alvi* and *G. apicola* including all taxa presented in Figures 3C and 3D. (A) Topologies of single gene trees at the basal node of the *S. alvi* lineage. (B) Topologies of single gene trees for the clade of the four closely related genomes of *S. alvi*. (C) Topologies of single gene trees at the node determining the relationship between the lineages of genomes of *G. apicola* from honey bee and bumble bee. For (A) and (C), genes which did not conform to one of the presented topologies are summarized in the last category indicated by a collapsed clade (triangle). For (B), five genes did not reveal the four closely related strains to be monophyletic. Therefore only 109 of the 114 conserved gene trees (96%) were included in the analysis.
(TIF)

Figure S7 Intragenic recombination detected with the program Geneconv between pairs of (A) *S. alvi* genomes and (B) *G. apicola* genomes. All shared genes of SAGs and reference genomes were analyzed (239 genes for *S. alvi* and 400 genes for *G. apicola*). Numbers of genes for which intragenic recombination was detected are indicated in red color. Total number of genes with evidence for intragenic recombination is given in absolute and relative values for each genome. Average fragment length of all recombination events between a given pair is shown in green color. Dendograms show the phylogenetic relationship between strains.
(TIF)

Figure S8 Distribution of pairwise *d*S values between the genomes of *S. alvi* wkB1 and B02. Compared to I20 versus P17 (Figure 4B), these two SAGs reveal uniform *d*S values. Colors indicate different ranges of *d*S values with yellow for *d*S<0.1, orange *d*S≥0.1, and red for *d*S≥1. Y-axis shows number of genes.
(TIF)

Table S1 Genotypes of 126 SAGs based on 16S rRNA gene amplicon sequencing and results of BLASTN analysis.
(PDF)

Table S2 Genes conflicting with overall patterns of divergence between O02, J21, and P14.
(PDF)

Table S3 Ratios (r/m) of recombination (r) and mutation (m) for different strains of *G. apicola* and *S. alvi* based on ClonalFrame analyses.
(PDF)
