# Supplementary figures and images for: Hidden Diversity in Honey Bee Gut Symbionts Detected by Single-Cell Genomics
Source: PLoS Genet. 2014 Sep 11;10(9):e1004596. doi: 10.1371/journal.pgen.1004596 (PMC4161309; doi:10.1371/journal.pgen.1004596)

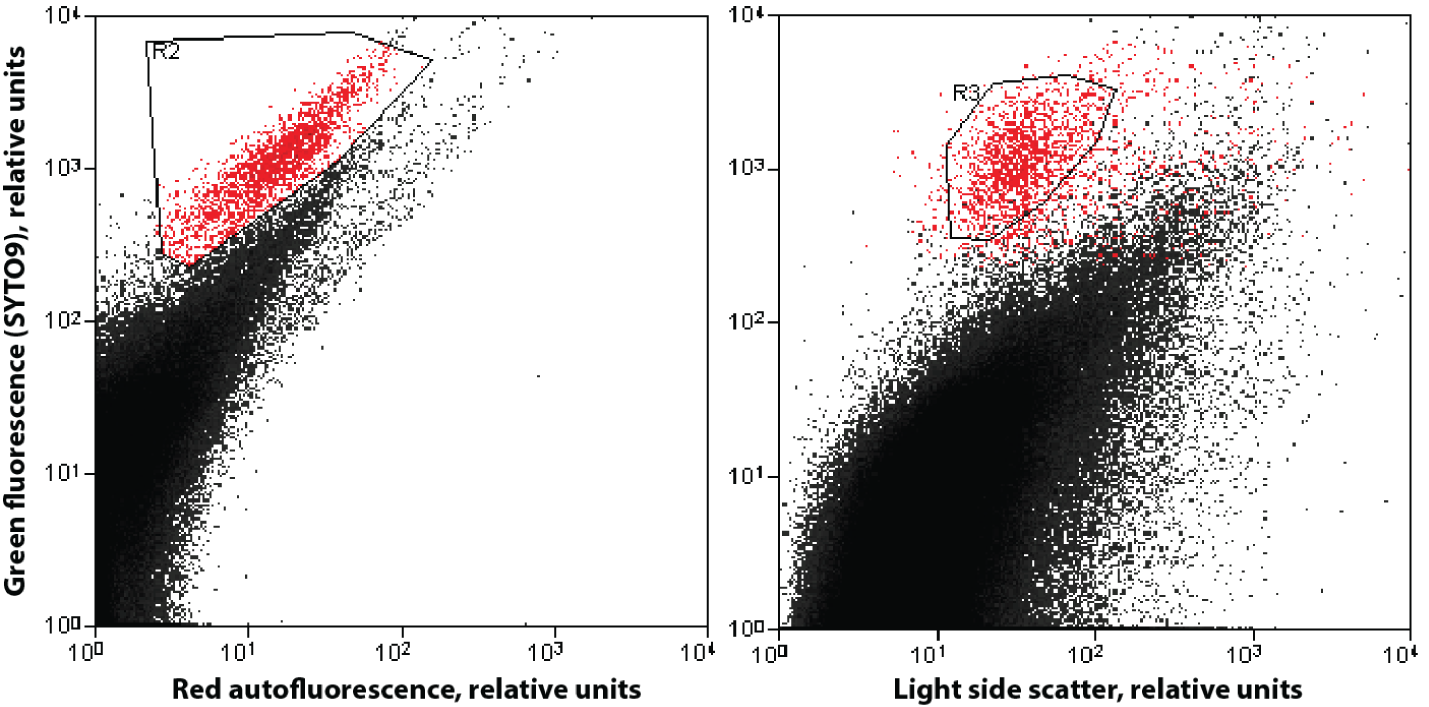

Supplement: Figure S1 — Flow cytometric dot plot of honey bee gut homogenate labeled with SYTO 9 for DNA. A combination of regions R2 and R3 were employed to separate bacterial cells from other particles. (TIF) [file pgen.1004596.s001.tif]

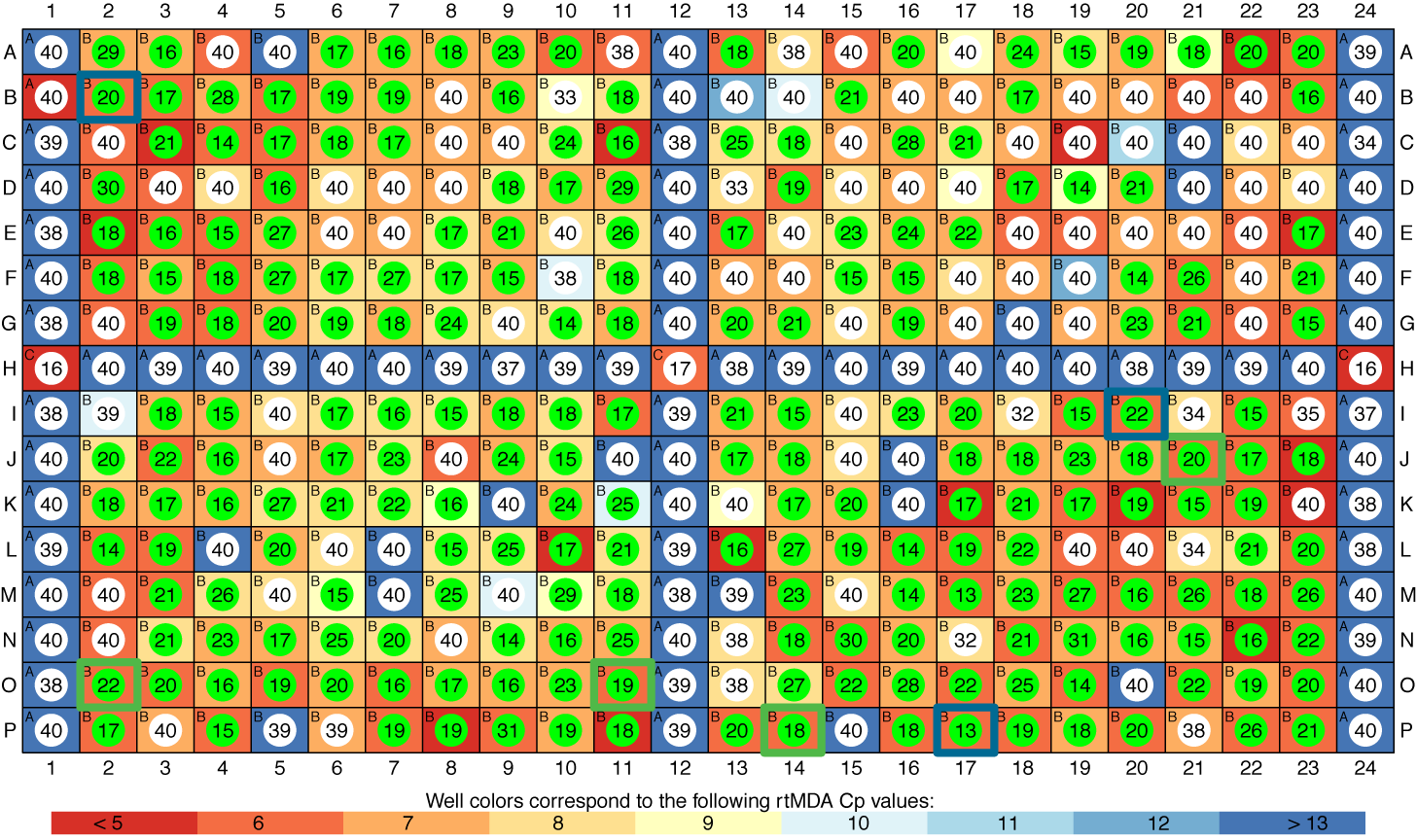

Supplement: Figure S2 — Reaction kinetics summary of real-time multiple displacement amplification (rtMDA) and quantitative PCR (qPCR) of the 16S rRNA gene for single cells sorted into a 384-well microplate. “A” indicates wells with no cells deposited (negative controls); “B” indicates wells (315 in total) with individual cells; and “C” indicates wells with 10 cells (positive controls). Well colors indicate real-time PCR critical point (Cp) values, i.e. the time required to produce half of the maximal fluorescence of the SYTO9 DNA stain during the rtMDA reaction. Green circle colors in wells indicate positive qPCR reactions with Ct values (numbers in circle) significantly lower than Ct values of negative controls. Single cells of S. alvi and G. apicola selected for whole-genome sequencing are highlighted with green and blue colored frames, respectively. (TIF) [file pgen.1004596.s002.tif]

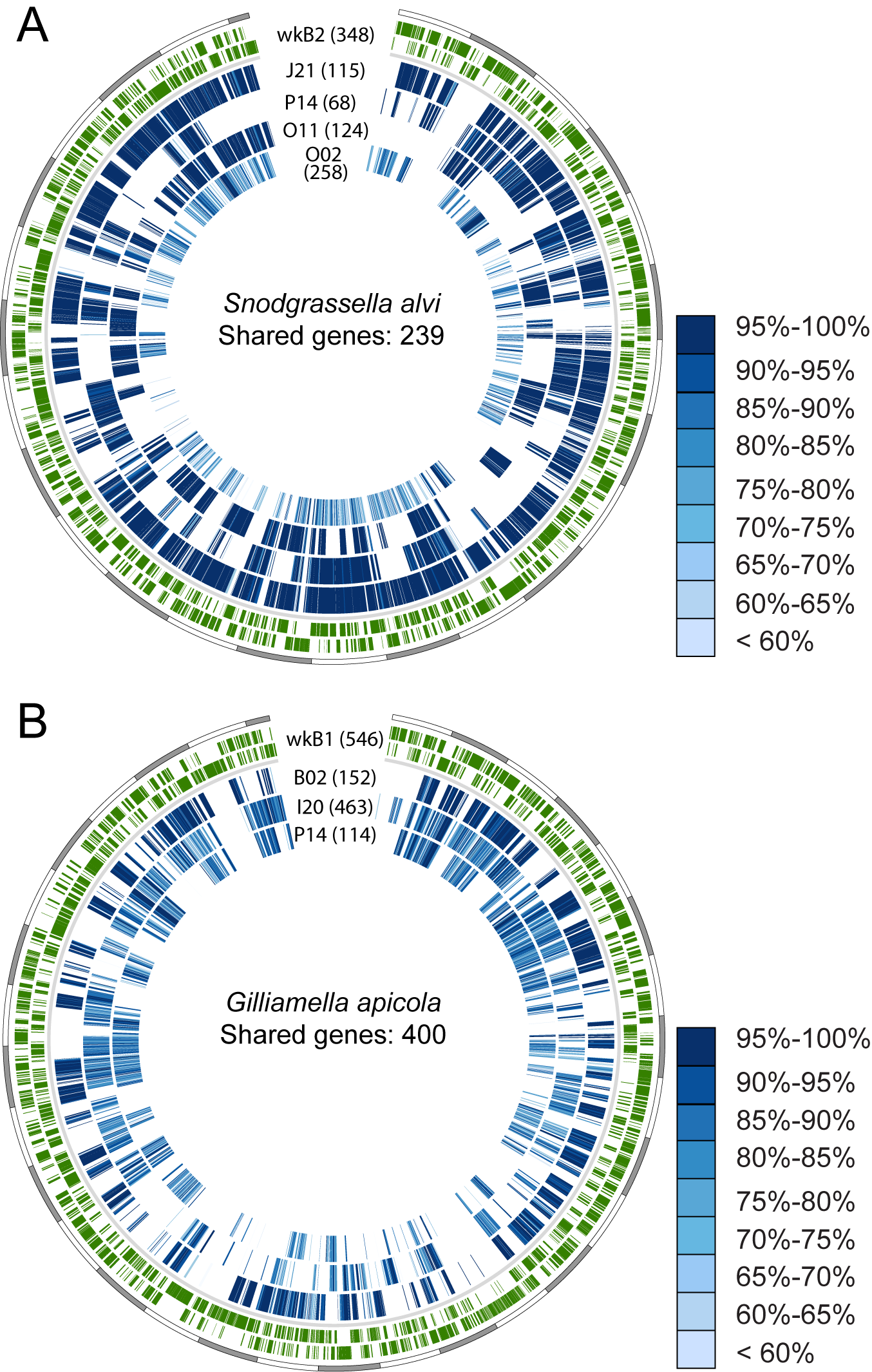

Supplement: Figure S3 — Mapping of orthologous genes of SAGs onto the reference genome of (A) S. alvi wkB2 and (B) G. apicola wkB1. Starting from outside, the first circle shows the scale of the reference genome representation in grey- and white-colored steps of 100 kb. The second and third circles (green color) depict the genes on the plus and minus strand of the reference genome. The blue circles represent genes of each SAG for which an ortholog has been identified in the reference genome. The blue color range denotes protein identity between SAG and reference genome according to the scale next to the genome circle. Note the differences in protein identities between different SAGs and reference genome reflecting the high variation in sequence divergence within S. alvi and G. apicola. Numbers in parentheses denote genome-specific genes neither shared with the reference nor with any other sequenced SAG of the same species (see also Figure S4). (TIF) [file pgen.1004596.s003.tif]

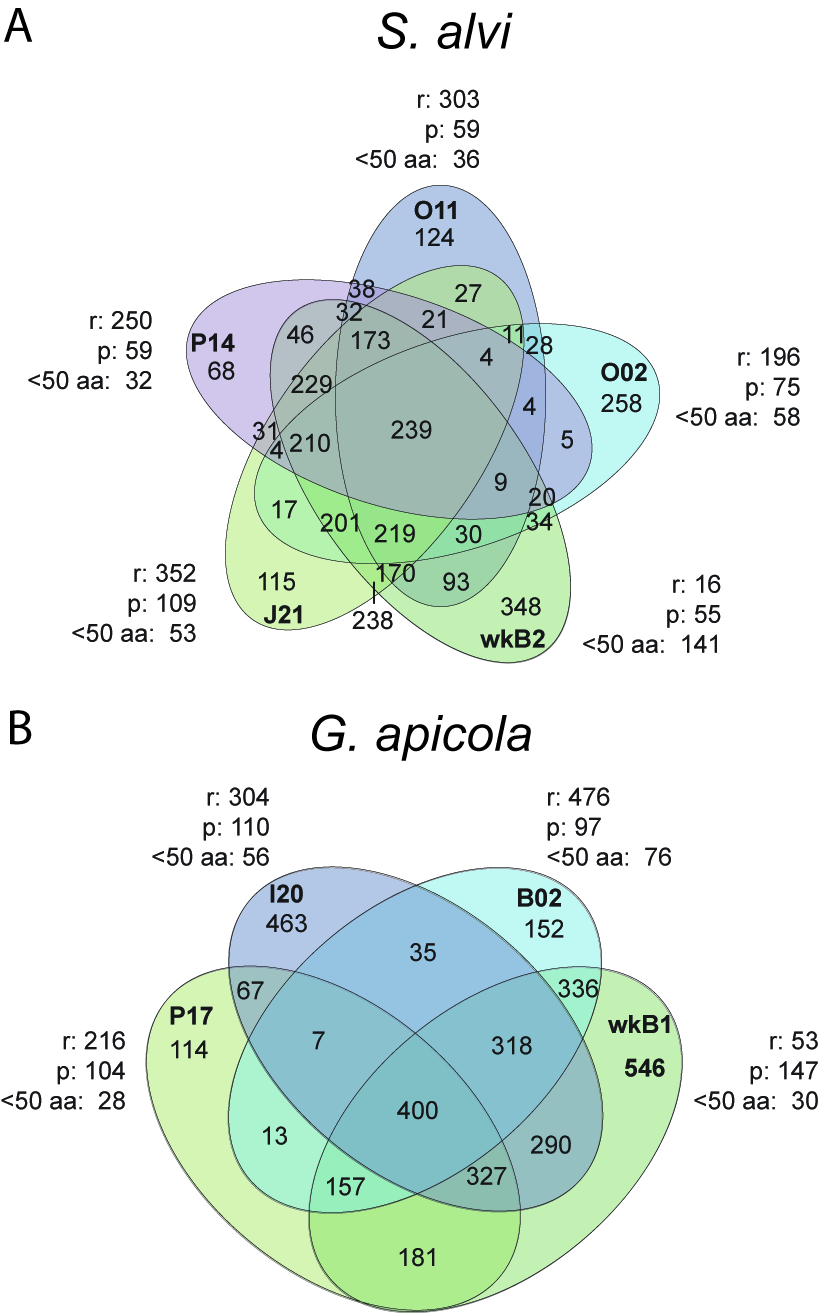

Supplement: Figure S4 — Venn diagrams showing number of orthologs between SAGs and reference genome for (A) S. alvi and (B) G. apicola. Remnants (r, genes which have partial hits to other genes), paralogs (p), and small genes (<50 aa, potential false positives) were subtracted from the number of genome-specific genes. (TIF) [file pgen.1004596.s004.tif]

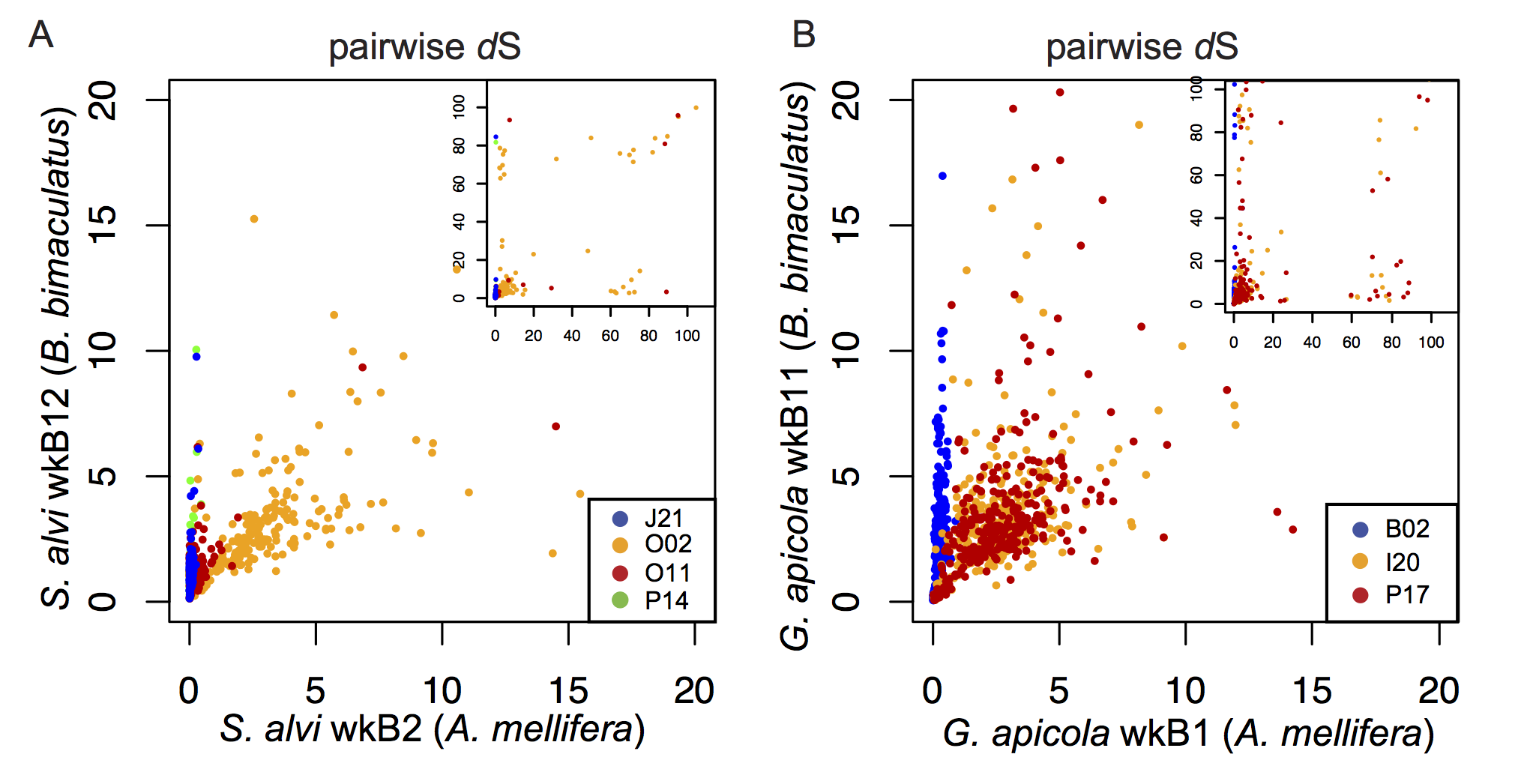

Supplement: Figure S5 — Plots show sequence divergence at synonymous sites (dS) of core genes for (A) S. alvi SAGs and (B) G. apicola SAGs (as shown in Figure 3, but on larger scales). Genes with dS values ≥3 can be considered at saturation due to the four possible bases in the genetic code. Inset shows all genes in one plot including those with unrealistically high dS values. (TIF) [file pgen.1004596.s005.tif]

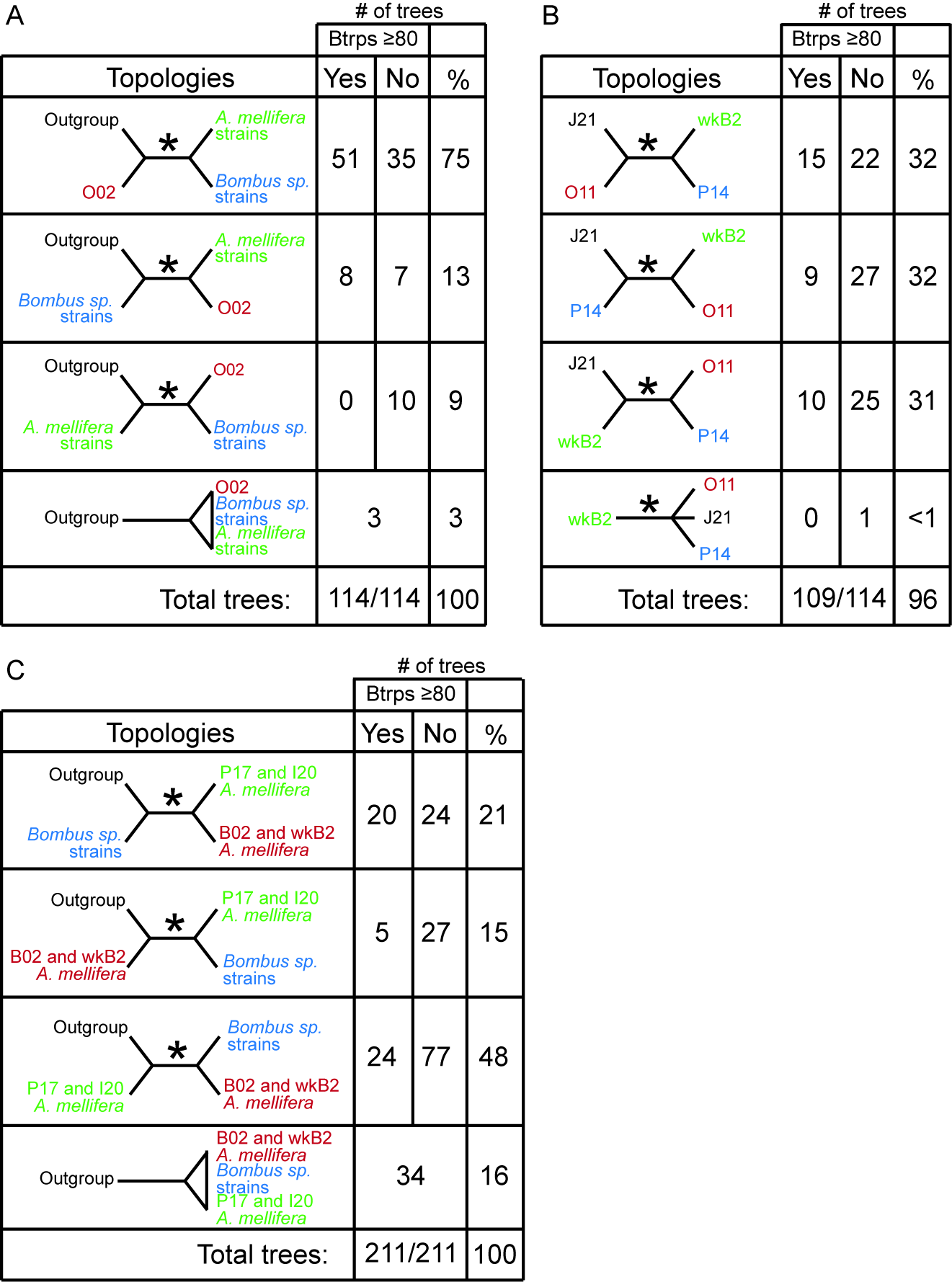

Supplement: Figure S6 — Summary of single gene tree topologies. Possible topologies are indicated by quartet representations. Values present the number of trees congruent with the depicted topology at the node indicated by an asterisk, divided into trees with and without Bootstrap (Btrps) support of ≥80. Relative values are also given (%). Data was extracted from 114 and 211 single gene trees of S. alvi and G. apicola including all taxa presented in Figures 3C and 3D. (A) Topologies of single gene trees at the basal node of the S. alvi lineage. (B) Topologies of single gene trees for the clade of the four closely related genomes of S. alvi. (C) Topologies of single gene trees at the node determining the relationship between the lineages of genomes of G. apicola from honey bee and bumble bee. For (A) and (C), genes which did not conform to one of the presented topologies are summarized in the last category indicated by a collapsed clade (triangle). For (B), five genes did not reveal the four closely related strains to be monophyletic. Therefore only 109 of the 114 conserved gene trees (96%) were included in the analysis. (TIF) [file pgen.1004596.s006.tif]

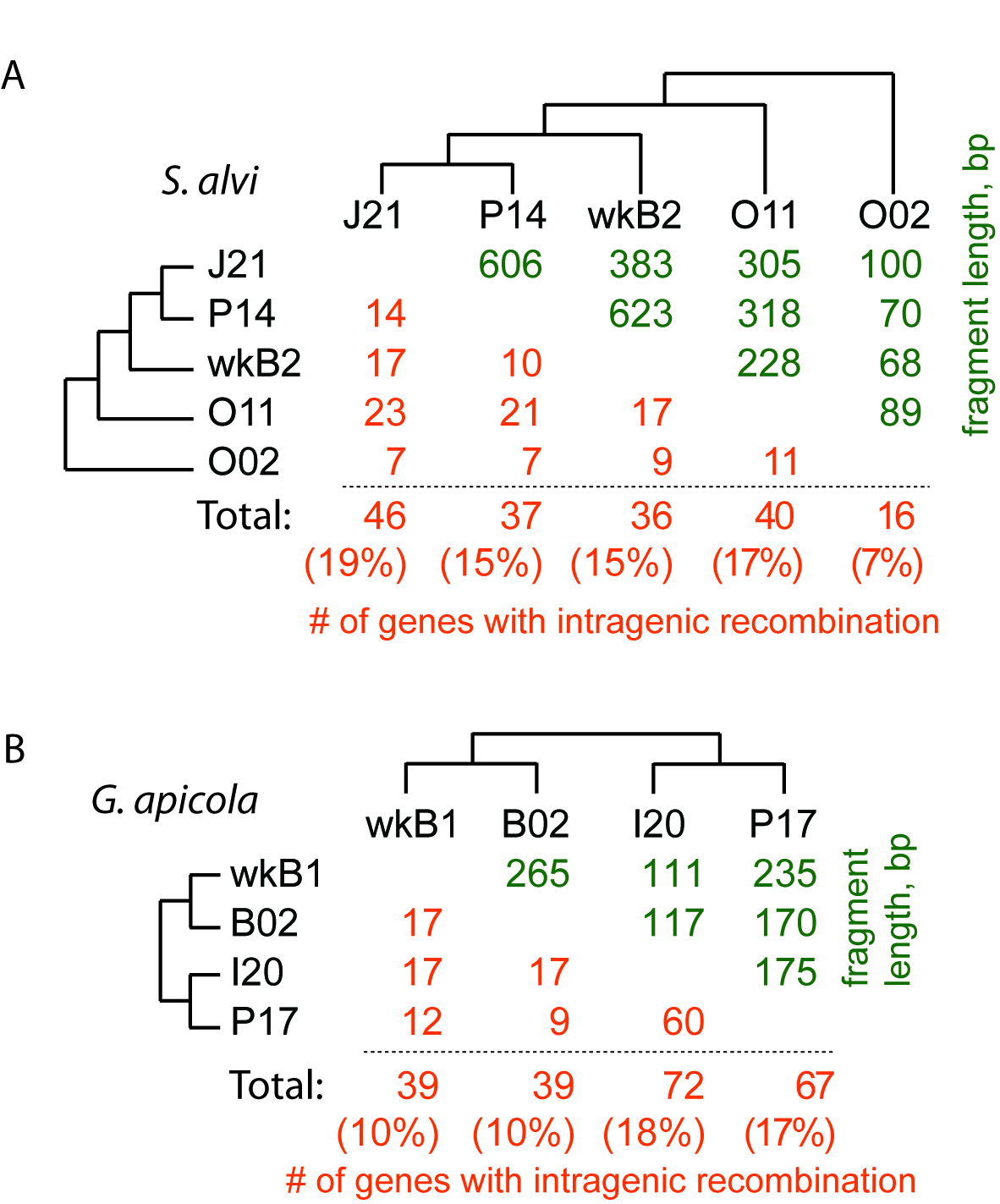

Supplement: Figure S7 — Intragenic recombination detected with the program Geneconv between pairs of (A) S. alvi genomes and (B) G. apicola genomes. All shared genes of SAGs and reference genomes were analyzed (239 genes for S. alvi and 400 genes for G. apicola). Numbers of genes for which intragenic recombination was detected are indicated in red color. Total number of genes with evidence for intragenic recombination is given in absolute and relative values for each genome. Average fragment length of all recombination events between a given pair is shown in green color. Dendograms show the phylogenetic relationship between strains. (TIF) [file pgen.1004596.s007.tif]

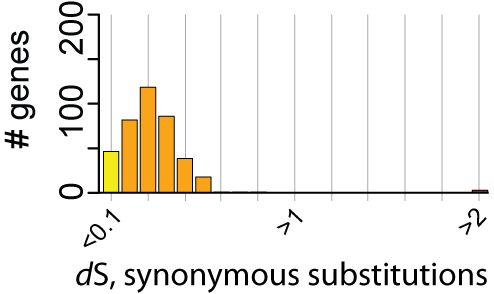

Supplement: Figure S8 — Distribution of pairwise dS values between the genomes of S. alvi wkB1 and B02. Compared to I20 versus P17 (Figure 4B), these two SAGs reveal uniform dS values. Colors indicate different ranges of dS values with yellow for dS<0.1, orange dS≥0.1, and red for dS≥1. Y-axis shows number of genes. (TIF) [file pgen.1004596.s008.tif]
