# Supplementary material for: Hidden Diversity in Honey Bee Gut Symbionts Detected by Single-Cell Genomics
Source: PLoS Genet. 2014 Sep 11;10(9):e1004596. doi: 10.1371/journal.pgen.1004596 (PMC4161309; doi:10.1371/journal.pgen.1004596)
Supplement: Table S1 — Genotypes of 126 SAGs based on 16S rRNA gene amplicon sequencing and results of BLASTN analysis. (PDF) [file pgen.1004596.s009.pdf]

Table S1. Genotypes of 126 SAGs based on 16S rRNA gene amplicon sequencing and results of BLASTN analysis.

		nr best BLASTN hit			Reference species BLASTN hit[b]	
Sample	16S rRNA [bp][a]	Identity [%]	Accession	Species in nr database	Species name	Identity [%]
A03	1353	99.85	JQ746651	*Snodgrassella alvi*	*Snodgrassella alvi* wkB2	99.56
A06	1357	99.71	JQ746651	*Snodgrassella alvi*	*Snodgrassella alvi* wkB2	99.41
A07	1352	99.85	JQ746651	*Snodgrassella alvi*	*Snodgrassella alvi* wkB2	99.56
A08	1336	99.70	EF187247	*Gilliamella apicola*	*Gilliamella apicola* wkB1	99.18
A09	1221	99.59	HM113176	*Gilliamella apicola*	*Gilliamella apicola* wkB1	98.94
A13	1357	100.00	JQ673239	*Snodgrassella alvi*	*Snodgrassella alvi* wkB2	99.48
A15	1346	99.70	JX878306	*Frischella perrara*	*Frischella perrara* PEB0191	99.70
A16	1366	99.85	JQ746651	*Snodgrassella alvi*	*Snodgrassella alvi* wkB2	99.56
A18	1313	99.62	HM113221	*Snodgrassella alvi*	*Snodgrassella alvi* wkB2	99.62
A22	624	98.08	HE613291	*Acetobacteriaceae sp.*	n.d.	n.d.
A23	1349	99.93	JQ746650	*Snodgrassella alvi*	*Snodgrassella alvi* wkB2	99.93
B02	1538	99.22	JQ936674	*Gilliamella apicola*	*Gilliamella apicola* wkB1	99.22
B03	1326	99.77	EF187247	*Gilliamella apicola*	*Gilliamella apicola* wkB1	99.25
B05	1354	99.85	JQ746651	*Snodgrassella alvi*	*Snodgrassella alvi* wkB2	99.56
B06	1310	99.47	JQ936674	*Gilliamella apicola*	*Gilliamella apicola* wkB1	99.47
B09	1391	99.64	JQ746651	*Snodgrassella alvi*	*Snodgrassella alvi* wkB2	99.35
B11	1361	99.93	JQ673239	*Snodgrassella alvi*	*Snodgrassella alvi* wkB2	99.41
B15	1360	99.27	JQ746650	*Snodgrassella alvi*	*Snodgrassella alvi* wkB2	99.27
B18	1355	100.00	JQ746651	*Snodgrassella alvi*	*Snodgrassella alvi* wkB2	99.71
B23	1356	99.78	JQ746650	*Snodgrassella alvi*	*Snodgrassella alvi* wkB2	99.78
C02	1320	99.55	EF187247	*Gilliamella apicola*	*Gilliamella apicola* wkB1	99.02
C04	1534	99.80	JX878306	*Frischella perrara*	*Frischella perrara* PEB0191	99.80
C06	1323	100.00	JQ746650	*Snodgrassella alvi*	*Snodgrassella alvi* wkB2	100.00
C08	1349	100.00	JQ746651	*Snodgrassella alvi*	*Snodgrassella alvi* wkB2	99.70
C10	624	100.00	JQ582002	*Gilliamella apicola*	*Gilliamella apicola* wkB1	99.68
C11	1276	99.76	EF187247	*Gilliamella apicola*	*Gilliamella apicola* wkB1	99.29
C14	1368	100.00	JQ673239	*Snodgrassella alvi*	*Snodgrassella alvi* wkB2	99.49
C17	1291	99.92	JQ673239	*Snodgrassella alvi*	*Snodgrassella alvi* wkB2	99.46
C23	1332	99.92	HM111936	*Gilliamella apicola*	*Gilliamella apicola* wkB1	99.47
D05	1358	99.78	JQ673244	*Gilliamella apicola*	*Gilliamella apicola* wkB1	99.41
D10	1367	99.56	EF187247	*Gilliamella apicola*	*Gilliamella apicola* wkB1	99.12
D14	1370	99.56	JQ673244	*Gilliamella apicola*	*Gilliamella apicola* wkB1	98.91
D16	1162	99.83	JQ673234	*Gilliamella apicola*	*Gilliamella apicola* wkB1	99.40
D18	1357	100.00	JQ673239	*Snodgrassella alvi*	*Snodgrassella alvi* wkB2	99.48
D23	1377	99.49	EF187247	*Gilliamella apicola*	*Gilliamella apicola* wkB1	98.98
E04	1343	99.70	EF187248	*Gilliamella apicola*	*Gilliamella apicola* wkB1	99.48
E06	1357	100.00	JQ673239	*Snodgrassella alvi*	*Snodgrassella alvi* wkB2	99.48
E08	589	97.79	JQ581781	*Gilliamella apicola*	*Gilliamella apicola* wkB1	96.94
E13	1321	99.77	EF187248	*Gilliamella apicola*	*Gilliamella apicola* wkB1	99.39
E15	624	99.52	JQ746651	*Snodgrassella alvi*	*Snodgrassella alvi* wkB2	98.88
E16	1318	99.92	JQ746651	*Snodgrassella alvi*	*Snodgrassella alvi* wkB2	99.62
E17	1280	99.69	EF187247	*Gilliamella apicola*	*Gilliamella apicola* wkB1	99.14
E21	1316	99.77	EF187247	*Gilliamella apicola*	*Gilliamella apicola* wkB1	99.24
F02	636	98.41	HE613310	*Lactobacillus sp.*	n.d.	n.d.
F02	1354	100.00	JQ673239	*Snodgrassella alvi*	*Snodgrassella alvi* wkB2	99.48
F06	1351	99.93	JQ673239	*Snodgrassella alvi*	*Snodgrassella alvi* wkB2	99.41
F08	1377	99.64	EF187247	*Gilliamella apicola*	*Gilliamella apicola* wkB1	99.13
F09	624	98.88	JQ746651	*Snodgrassella alvi*	*Snodgrassella alvi* wkB2	98.24
F11	1369	99.78	EF187247	*Gilliamella apicola*	*Gilliamella apicola* wkB1	99.27
F14	1325	100.00	JQ746650	*Snodgrassella alvi*	*Snodgrassella alvi* wkB2	100.00
F16	1361	99.93	JQ746650	*Snodgrassella alvi*	*Snodgrassella alvi* wkB2	99.93
F20	1366	99.71	JQ746651	*Snodgrassella alvi*	*Snodgrassella alvi* wkB2	99.41
G03	1333	100.00	JQ673239	*Snodgrassella alvi*	*Snodgrassella alvi* wkB2	99.48
G06	624	99.68	JQ581781	*Gilliamella apicola*	*Gilliamella apicola* wkB1	98.72
G07	620	99.35	JQ581772	*Gilliamella apicola*	*Gilliamella apicola* wkB1	98.71
G10	1320	99.70	EF187247	*Gilliamella apicola*	*Gilliamella apicola* wkB1	99.17
G11	1359	99.71	EF187248	*Gilliamella apicola*	*Gilliamella apicola* wkB1	99.48
G14	1346	100.00	JQ673239	*Snodgrassella alvi*	*Snodgrassella alvi* wkB2	99.48
G15	1322	99.62	HM113221	*Snodgrassella alvi*	*Snodgrassella alvi* wkB2	99.62
G16	620	99.19	JQ581835	*Snodgrassella alvi*	*Snodgrassella alvi* wkB2	97.75
G20	1277	99.69	JQ673249	*Gilliamella apicola*	*Gilliamella apicola* wkB1	99.06
G21	1361	99.93	JQ746650	*Snodgrassella alvi*	*Snodgrassella alvi* wkB2	99.93

		nr best BLASTN hit			Reference species BLASTN hit[b]	
Sample	16S rRNA [bp][a]	Identity [%]	Accession	Species in nr database	Species name	Identity [%]
I03	1305	99.69	EF187247	*Gilliamella apicola*	*Gilliamella apicola* wkB1	99.16
I04	1320	98.18	EF187250	*Frischella perrara*	*Frischella perrara* PEB0191	98.11
I07	1344	99.70	JQ673249	*Gilliamella apicola*	*Gilliamella apicola* wkB1	99.03
I09	1377	99.64	EF187247	*Gilliamella apicola*	*Gilliamella apicola* wkB1	99.20
I10	1328	99.55	JQ936674	*Gilliamella apicola*	*Gilliamella apicola* wkB1	99.55
I11	1365	99.56	JQ936674	*Gilliamella apicola*	*Gilliamella apicola* wkB1	99.56
I17	1357	99.78	JX878306	*Frischella perrara*	*Frischella perrara* PEB0191	99.78
I19	1372	99.42	JQ936674	*Gilliamella apicola*	*Gilliamella apicola* wkB1	99.42
I20	1538	99.09	JQ936674	*Gilliamella apicola*	*Gilliamella apicola* wkB1	99.09
I22	1534	99.93	JX878306	*Frischella perrara*	*Frischella perrara* PEB0191	99.93
J02	1319	99.47	AY370192	*Gilliamella apicola*	*Gilliamella apicola* wkB1	99.32
J03	1334	99.70	EF187248	*Gilliamella apicola*	*Gilliamella apicola* wkB1	99.48
J04	1371	99.93	JQ673239	*Snodgrassella alvi*	*Snodgrassella alvi* wkB2	99.42
J06	1297	99.92	HM111936	*Gilliamella apicola*	*Gilliamella apicola* wkB1	99.46
J08	1379	99.71	EF187248	*Gilliamella apicola*	*Gilliamella apicola* wkB1	99.27
J10	619	96.28	JQ746651	*Snodgrassella alvi*	*Snodgrassella alvi* wkB2	95.65
J17	619	100.00	JQ581810	*Gilliamella apicola*	*Gilliamella apicola* wkB1	99.03
J19	679	100.00	JQ581937	*Snodgrassella alvi*	*Snodgrassella alvi* wkB2	99.85
J21	1548	99.68	JQ746650	*Snodgrassella alvi*	*Snodgrassella alvi* wkB2	99.68
J22	610	99.67	JQ581780	*Gilliamella apicola*	*Gilliamella apicola* wkB1	98.52
J23	1369	99.63	JQ746650	*Snodgrassella alvi*	*Snodgrassella alvi* wkB2	99.63
K02	1306	99.69	JQ746651	*Snodgrassella alvi*	*Snodgrassella alvi* wkB2	99.39
K06	1332	99.62	HM113280	*Gilliamella apicola*	*Gilliamella apicola* wkB1	99.33
K10	610	100.00	JQ582002	*Gilliamella apicola*	*Gilliamella apicola* wkB1	99.67
K15	610	99.18	JQ581937	*Snodgrassella alvi*	*Snodgrassella alvi* wkB2	99.02
K17	608	96.55	JQ581810	*Gilliamella apicola*	*Gilliamella apicola* wkB1	95.57
K18	1280	99.77	EF187248	*Gilliamella apicola*	*Gilliamella apicola* wkB1	99.30
K18	608	99.84	JQ581772	*Gilliamella apicola*	*Gilliamella apicola* wkB1	99.18
K19	1332	99.47	JQ673239	*Snodgrassella alvi*	*Snodgrassella alvi* wkB2	99.10
K21	1361	99.56	JQ936674	*Gilliamella apicola*	*Gilliamella apicola* wkB1	99.56
K22	1321	99.77	JQ673249	*Gilliamella apicola*	*Gilliamella apicola* wkB1	99.47
L2	1359	99.56	JQ936674	*Gilliamella apicola*	*Gilliamella apicola* wkB1	99.56
L10	609	99.84	JQ581772	*Gilliamella apicola*	*Gilliamella apicola* wkB1	99.18
L11	1353	99.70	EF187247	*Gilliamella apicola*	*Gilliamella apicola* wkB1	99.26
L15	1276	99.84	EF187247	*Gilliamella apicola*	*Gilliamella apicola* wkB1	99.29
L16	1352	99.70	EF187247	*Gilliamella apicola*	*Gilliamella apicola* wkB1	99.26
L22	1305	99.92	JQ746651	*Snodgrassella alvi*	*Snodgrassella alvi* wkB2	99.62
L23	1324	99.62	JQ746650	*Gilliamella apicola*	*Snodgrassella alvi* wkB2	99.62
M16	1361	99.41	EF187248	*Gilliamella apicola*	*Gilliamella apicola* wkB1	99.04
M18	1333	100.00	JQ673239	*Snodgrassella alvi*	*Snodgrassella alvi* wkB2	99.48
M20	1319	99.85	EF187247	*Gilliamella apicola*	*Gilliamella apicola* wkB1	99.32
N04	1271	99.76	EF187247	*Gilliamella apicola*	*Gilliamella apicola* wkB1	99.21
N05	1347	99.85	JQ746651	*Snodgrassella alvi*	*Snodgrassella alvi* wkB2	99.55
N06	636	99.84	JQ581967	*Snodgrassella alvi*	*Snodgrassella alvi* wkB2	99.68
N10	639	100.00	JQ746651	*Snodgrassella alvi*	*Snodgrassella alvi* wkB2	99.38
N11	1276	99.84	EF187247	*Gilliamella apicola*	*Gilliamella apicola* wkB1	99.29
N16	1374	99.93	JQ746650	*Snodgrassella alvi*	*Snodgrassella alvi* wkB2	99.93
N18	1324	99.85	EF187247	*Gilliamella apicola*	*Gilliamella apicola* wkB1	99.47
N22	1352	99.85	JQ746651	*Snodgrassella alvi*	*Snodgrassella alvi* wkB2	99.56
N23	1282	99.61	HM113221	*Snodgrassella alvi*	*Snodgrassella alvi* wkB2	99.61
O02	1547	99.55	JQ746651	*Snodgrassella alvi*	*Snodgrassella alvi* wkB2	99.29
O03	609	99.67	JQ581770	*Gilliamella apicola*	*Gilliamella apicola* wkB1	98.52
O05	609	98.69	JQ581835	*Snodgrassella alvi*	*Snodgrassella alvi* wkB2	97.55
O06	1276	99.92	JQ673234	*Gilliamella apicola*	*Gilliamella apicola* wkB1	99.14
O09	1368	99.71	EF187247	*Gilliamella apicola*	*Gilliamella apicola* wkB1	99.20
O10	1279	100.00	JQ673252	*Snodgrassella alvi*	*Snodgrassella alvi* wkB2	99.45
O11	1547	99.81	JQ746651	*Snodgrassella alvi*	*Snodgrassella alvi* wkB2	99.55
O15	614	99.84	JQ581781	*Gilliamella apicola*	*Gilliamella apicola* wkB1	98.86
O19	1355	99.70	EF187247	*Gilliamella apicola*	*Gilliamella apicola* wkB1	99.19
O22	1349	99.63	JQ746650	*Snodgrassella alvi*	*Snodgrassella alvi* wkB2	99.63
P04	1360	99.56	JQ936674	*Gilliamella apicola*	*Gilliamella apicola* wkB1	99.56
P14	1547	99.74	JQ746651	*Snodgrassella alvi*	*Snodgrassella alvi* wkB2	99.48
P16	1353	99.85	JQ746651	*Snodgrassella alvi*	*Snodgrassella alvi* wkB2	99.56
P17	1538	98.96	JQ936674	*Gilliamella apicola*	*Gilliamella apicola* wkB1	98.96

[a]length of sequenced 16S rRNA fragment; [b]BLASTN hit against the typing strain for described species
