# Supplementary material for: Hidden Diversity in Honey Bee Gut Symbionts Detected by Single-Cell Genomics
Source: PLoS Genet. 2014 Sep 11;10(9):e1004596. doi: 10.1371/journal.pgen.1004596 (PMC4161309; doi:10.1371/journal.pgen.1004596)
Supplement: Table S2 — Genes conflicting with overall patterns of divergence between O02, J21, and P14. (PDF) [file pgen.1004596.s010.pdf]

Table S2. Genes conflicting with overall patterns of divergence between O02, J21, and P14.

Gene in wkB2	Annotation	Best BLASTX hit in nr	O02 - J21[a]		P14 - J21[a]		P14 - O02[a]	
			abs *dS*	rel *dS*	abs *dS*	rel *dS*	abs *dS*	rel. *dS*
SALWKB2_0167	ArsR family transcriptional regulator	Paenibacillus sp. Y412MC10; ref|WP_009593529.1|	0.13	0.43	0.05	0.16	0.13	0.42
SALWKB2_0168	Multidrug resistance protein	Lactobacillus rhamnosus LOCK900; ref|YP_008202154.1|	0.34	0.33	0.44	0.43	0.24	0.24
SALWKB2_0450	Urease accessory protein E	Haemophilus influenzae PittEE; ref|YP_001289941.1|	0.20	0.44	0.05	0.11	0.20	0.45
SALWKB2_0452	Urease accessory protein J	Pseudomonas sp. GM50; ref|WP_008009765.1|	0.10	0.41	0.05	0.21	0.09	0.38
SALWKB2_0453	Urease accessory protein G	Haemophilus influenzae; ref|WP_005692066.1|	0.05	0.51	0.02	0.23	0.03	0.27
SALWKB2_0454	Urease accessory protein D	Haemophilus paraphrohaemolyticus; ref|WP_005708164.1|	0.05	0.29	0.08	0.41	0.06	0.30
SALWKB2_0455	Conserved hypothetical protein; COG1673	Planctomyces limnophilus DSM 3776; ref|YP_003631252.1|	0.34	0.33	0.44	0.43	0.24	0.24
SALWKB2_1196	GMP synthase	Neisseria weaveri; ref|WP_004284982.1|	0.19	0.41	0.08	0.17	0.19	0.41
SALWKB2_1201	DNA repair rotein radC	Flavobacteriaceae bacterium HQM9; ref|WP_010522576.1|	0.19	0.38	0.12	0.25	0.18	0.36
SALWKB2_1202	LysR-type transcriptional regulator	Providencia burhodogranariea; ref|WP_008910958.1|	0.11	0.31	0.11	0.32	0.13	0.36
SALWKB2_1204	Efflux permease of the major facilitator superfamily	Providencia burhodogranariea; ref|WP_008910960.1|	0.05	0.21	0.10	0.43	0.08	0.36
SALWKB2_1206	General stress response protein CsbD	Lactobacillus fermentum; ref|WP_023465858.1|	0.31	0.45	0.18	0.25	0.21	0.30
SALWKB2_1294	Paraquat-inducible protein B	Neisseria shayeganii; ref|WP_009118480.1|	0.32	0.36	0.13	0.14	0.43	0.49

[a]absolute (abs) and relative (rel) *dS* values are shown for the three comparisons.
