# Supplementary material for: Hidden Diversity in Honey Bee Gut Symbionts Detected by Single-Cell Genomics
Source: PLoS Genet. 2014 Sep 11;10(9):e1004596. doi: 10.1371/journal.pgen.1004596 (PMC4161309; doi:10.1371/journal.pgen.1004596)
Supplement: Table S3 — Ratios (r/m) of recombination (r) and mutation (m) for different strains of G. apicola and S. alvi based on ClonalFrame analyses. (PDF) [file pgen.1004596.s011.pdf]

Table S3. Ratios (r/m) of recombination (r) and mutation (m) for different strains of *G. apicola* and *S. alvi* based on ClonalFrame analyses.

	**All substitutions considered**[a]		**Only substitutions with probabilities ≥0.95 considered**[b]					
	**Run 1**	**Run 2**	**Run 1**			**Run 1**		
**Strain**	**r / m**	**r / m**	**r / m**	**# recomb.**	**# mut.**	**r / m**	**# recomb.**	**# mut.**
G. apicola								
P17	5.1	5.1	15.7	5427	345	15.8	5551	351
I20	7.0	7.0	23.5	7769	330	23.8	7782	327
B02	0.5	0.5	0.8	776	931	0.8	788	933
wkB1	0.4	0.4	0.5	478	972	0.5	471	947
S. alvi								
J21	0.2	0.2	0.2	168	1083	0.2	198	1232
O02[c]	6.6	6.5	n.d.	0	0	n.d.	0	0
O11	1.1	1.1	0.4	301	820	0.3	288	823
P14	0.3	0.3	0.2	214	1407	0.2	249	1343
wkB2	0.2	0.2	0.2	270	1304	0.1	138	1177

[a] ratio r/m was calculated based on the sum of the probabilities of all substitutions to have occurred by recombination divided by the sum of the probabilities of all substitutions to have occurred by mutation.

[b] ratio r/m was calculated based on the sum of all positions for which the probability of substitution via mutation was ≥0.95 divided by the sum of all positions for which the probability of substitution via recombination was ≥0.95.

[c]O02 was too divergent to reliably estimate r/m values with ClonalFrame. Substitution saturation resulted in high r/m values, when all sites were considered. No r/m ratios (n.d., not determined) could be calculated when considering only substitutions with probabilities ≥0.95, because substitution probabilities were consistently <0.95.
